# Free fatty acid receptors: structural models and elucidation of ligand binding interactions

**DOI:** 10.1186/s12900-015-0044-2

**Published:** 2015-09-07

**Authors:** Irina G. Tikhonova, Elena Poerio

**Affiliations:** Molecular Therapeutics, School of Pharmacy, Medical Biology Centre, Queen’s University Belfast, Belfast, BT9 7BL Northern Ireland UK

## Abstract

**Background:**

The free fatty acid receptors (FFAs), including FFA1 (orphan name: GPR40), FFA2 (GPR43) and FFA3 (GPR41) are G protein-coupled receptors (GPCRs) involved in energy and metabolic homeostasis. Understanding the structural basis of ligand binding at FFAs is an essential step toward designing potent and selective small molecule modulators.

**Results:**

We analyse earlier homology models of FFAs in light of the newly published FFA1 crystal structure co-crystallized with TAK-875, an ago-allosteric ligand, focusing on the architecture of the extracellular binding cavity and agonist-receptor interactions. The previous low-resolution homology models of FFAs were helpful in highlighting the location of the ligand binding site and the key residues for ligand anchoring. However, homology models were not accurate in establishing the nature of all ligand-receptor contacts and the precise ligand-binding mode. From analysis of structural models and mutagenesis, it appears that the position of helices 3, 4 and 5 is crucial in ligand docking. The FFA1-based homology models of FFA2 and FFA3 were constructed and used to compare the FFA subtypes. From docking studies we propose an alternative binding mode for orthosteric agonists at FFA1 and FFA2, involving the interhelical space between helices 4 and 5. This binding mode can explain mutagenesis results for residues at positions 4.56 and 5.42. The novel FFAs structural models highlight higher aromaticity of the FFA2 binding cavity and higher hydrophilicity of the FFA3 binding cavity. The role of the residues at the second extracellular loop used in mutagenesis is reanalysed. The third positively-charged residue in the binding cavity of FFAs, located in helix 2, is identified and predicted to coordinate allosteric modulators.

**Conclusions:**

The novel structural models of FFAs provide information on specific modes of ligand binding at FFA subtypes and new suggestions for mutagenesis and ligand modification, guiding the development of novel orthosteric and allosteric chemical probes to validate the importance of FFAs in metabolic and inflammatory conditions. Using our FFA homology modelling experience, a strategy to model a GPCR, which is phylogenetically distant from GPCRs with the available crystal structures, is discussed.

**Electronic supplementary material:**

The online version of this article (doi:10.1186/s12900-015-0044-2) contains supplementary material, which is available to authorized users.

## Background

The free fatty acid receptors (FFAs) belong to Group A of the G protein-coupled receptor (GPCR) family and are activated endogenously by free fatty acids of different chain lengths with varying levels of specificity [1]. The free fatty acid receptor 1 (FFA1), previously known as GPR40, has a preference to bind the long-chain fatty acids with more than 12 carbon atoms [2, 3]. The free fatty acid receptor 2 (FFA2), previously known as GPR43, and the free fatty acid receptor 3 (FFA3), known as GPR41, respond to short-chain fatty acids that have less than 5 carbon atoms [4]. FFAs have more than 30 % sequence identity.

The recent discovery of FFAs involvement in glucose and lipid homeostasis, adiposity and inflammation has raised great interest to find small molecule ligands modulating the function of these receptors and probe them in the treatment of various metabolic and inflammatory conditions including obesity, type 2 diabetes, atherosclerosis, cardiovascular diseases, ulcerative colitis, Crohn’s disease and irritable bowel disease [5–8].

**Fig. 1:**
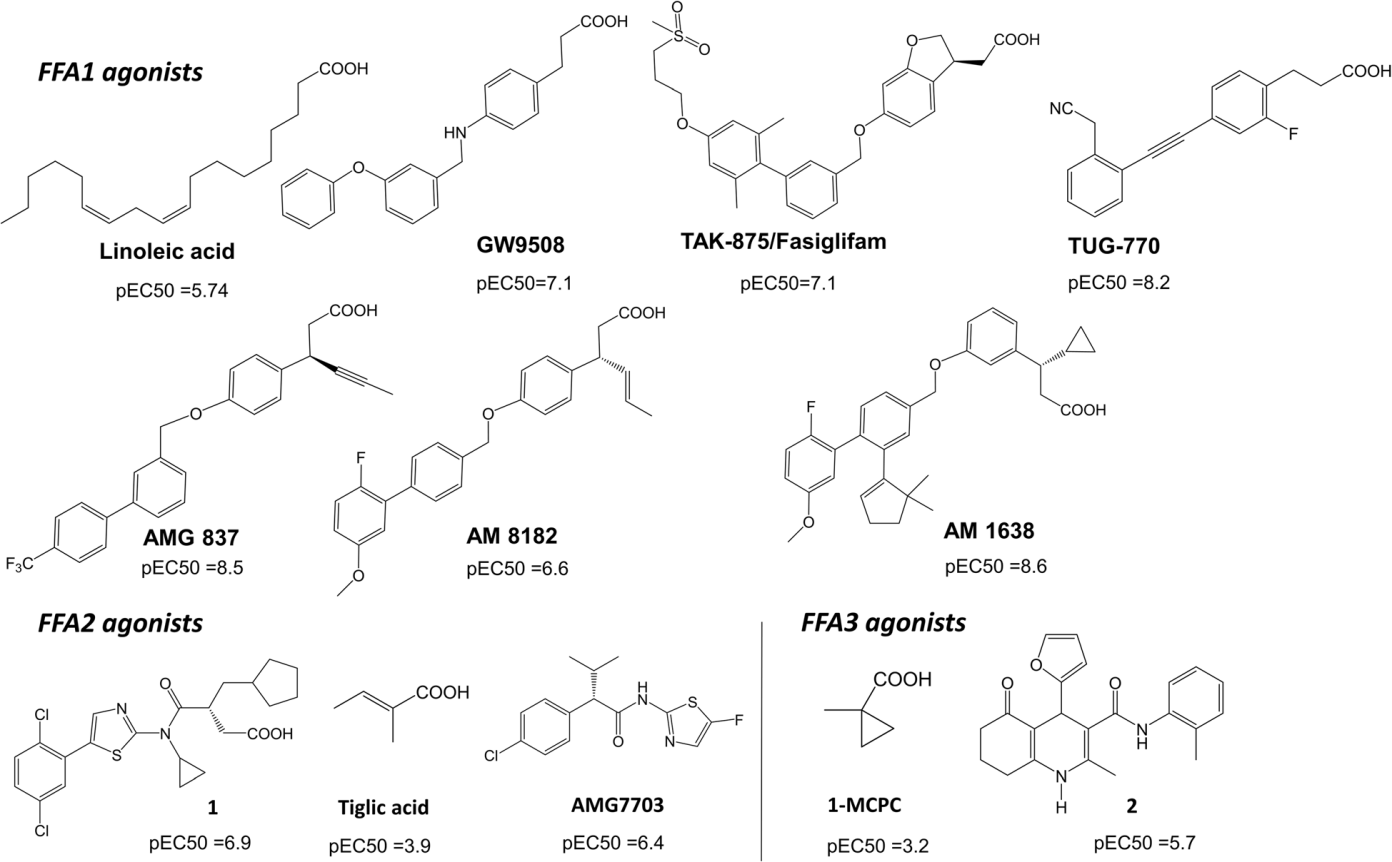
Agonists of the free fatty acid 1–3 receptors. The potency of compounds was taken from ref. 5, 9 and 13

Several FFA1 agonists including GW9508, TAK-875/fasiglifam, AMG-837, AM1638, AM8182 (Fig. 1), LY2881835, JTT-851 and P11187 were developed by industry using high-throughput screening and subsequent medicinal chemistry. Some of these agonists were tested in clinical trials but removed due to toxicity [9]. Small selective FFA2/FFA3 carboxylic acids derived from the endogenous fatty acids (Fig. 1) have been developed as FFA2/FFA3 agonists by academia, though with poor potency [10]. A series of synthetic agonists has been patented by Euroscreen with potency up to 13nM [11]. AMG7703, also referred to as 4-CMTB, is a FFA2 selective allosteric agonist (Fig. 1) that was discovered by Amgen to inhibit lipolysis [12], yet, unfavourable pharmacokinetic properties of AMG7703 prevented this compound from progressing to clinical trials [12]. No highly potent orthosteric agonists have been developed at FFA3 to date. A series of FFA3 selective molecules, for example **2** (Fig. 1) was reported by Arena Pharmaceuticals to act as positive or negative allosteric modulators [9]. It is evident that a few available ligands of FFAs have various limitations for clinical tests and the development of novel FFA ligands presenting drug-like properties is an emerging challenge to validate the role of FFAs modulation in the therapy of metabolic and immune disorders.

Understanding the structural basis of ligand binding at FFAs would benefit discovery of potent and selective small molecule modulators. Until recently, homology modelling in conjunction with mutagenesis has been used in non-direct identification of ligand interactions in FFAs [13, 14]. The structure-based approach using the validated FFA1 homology model together with ligand-based approaches have been probed in *in silico* search of orthosteric binders [15]. However, the crystal structure of FFA1 in complex with the ago-allosteric ligand, TAK-875 has been recently published [16], providing the first direct information of ligand binding at FFA1. In this work we first compare previous homology models of FFA1 [13, 14, 17] with the FFA1 crystallographic structure and use the experimental structure for docking of orthosteric and allosteric agonists to further delineate the agonist binding mode at FFA1. We next use the FFA1 structure to build novel homology models of FFA2 and FFA3 and compare the agonist binding site of the receptor subtypes. Throughout the study we link novel structural models of FFAs with available mutagenesis and structure-activity relationships (SAR) data. Our work extends the application of the recent FFA1 crystal structure, further predicts agonist binding regions at FFAs and suggests amino acid residues for mutagenesis to verify their role in binding of orthosteric and allosteric agonists. Our work provides general suggestions in modelling of distantly related GPCRs.

## Results and discussion

### Free fatty acid receptor 1

#### Structural models

**Fig. 2:**
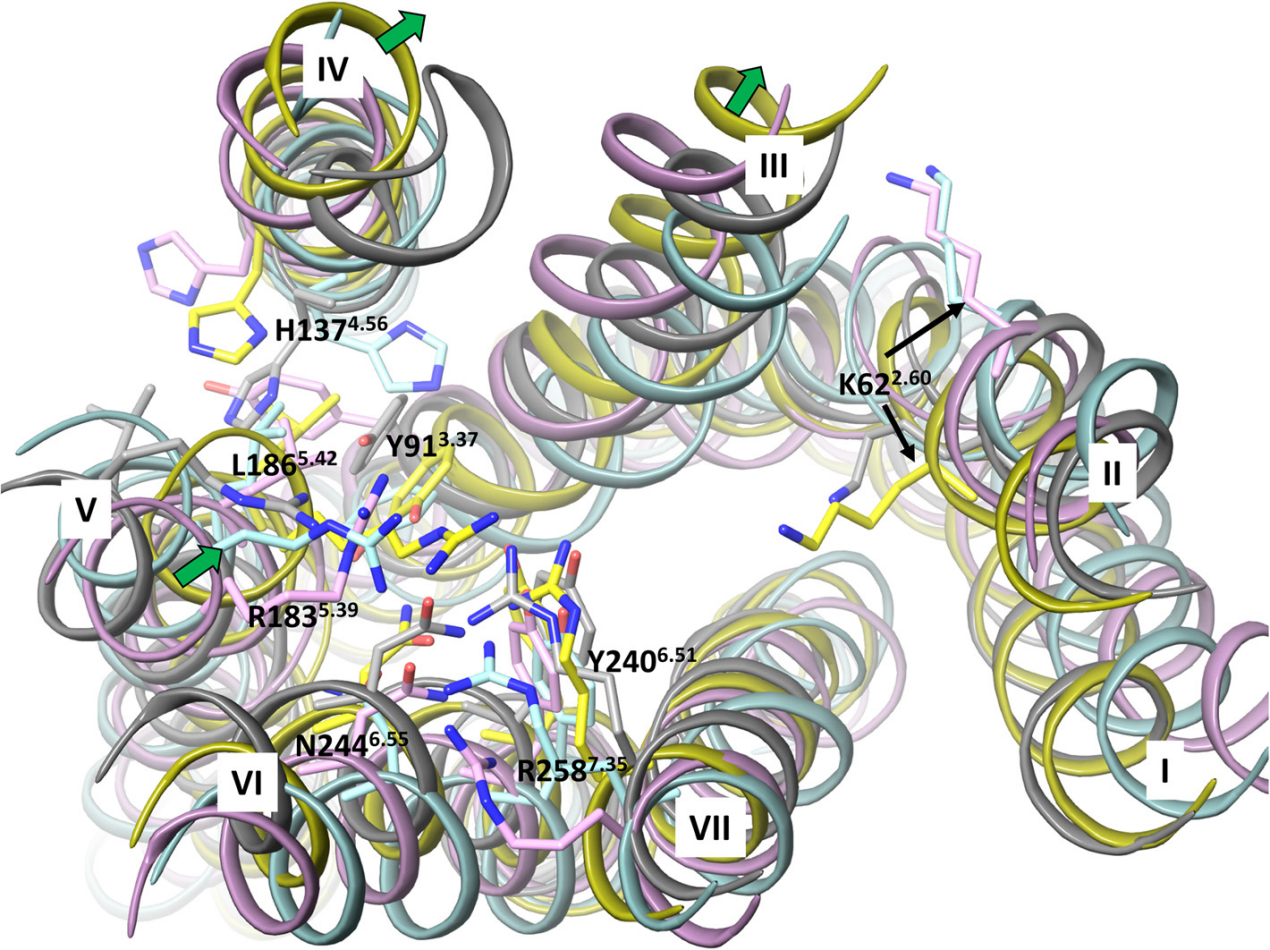
The superimposition of the FFA1 crystal structure and homology models base on the backbone of the helices in the extracellular side. The crystal structure, rhodopsin, β_2_ adrenergic and PAR_1_-based homology models are in yellow, cyan, pink and grey colour, respectively. Residues predicted to be important for ligand coordination based on mutagenesis and residue K62^2.60^, representing the possible anchoring point for allosteric ligands are shown in stick-like representation. The green arrows indicate the large movement of helices 3, 4 and 5 in the FFA1 crystal structure compared to the homology models

We evaluate the earlier homology models of FFA1 built by *Tikhonova et al*. [13, 15] and *Sum, et al.* [14, 17] based on rhodopsin and β_2_ adrenergic (β_2_) receptor crystal structures, templates with 16 % sequence identity to model the ligand binding site with the FFA1 crystal structure. In addition, the homology model based on the protease-activated receptor 1 (PAR_1_) crystal structure [18], the available template with the highest sequence identity, 26 % is constructed and included to the analysis. The backbone superimposition of helices of the crystal structure and homology models is shown in Fig. 2. The root-mean-square deviation (RMSD) value for the helix backbone is 2.8 Å for the rhodopsin-based and 1.9 Å for β_2_- and PAR_1_ -based FFA1 homology models. In the upper side of the helical bundle forming the ligand binding site a significant deviation is observed for helices 3, 4 and 5 with a backbone RMSD of 2–3.4 Å.

The earlier FFA1 homology models and mutagenesis [13, 14, 17, 19] have predicted that an agonist binding site is located between helices 3, 4, 5, 6 and 7, involving ^a^Y91^3.37^, H137^4.56^, R183^5.39^, L186^5.42^, Y240^6.51^, N244^6.55^ and R258^7.35^ [13, 14] (Fig. 2). The position and orientation of Y240^6.51^, N244^6.55^ and R258^7.35^ are similar between modelled and crystal structures with a RMSD approaching 1.6 Å. In contrast, the RMSD of Y91^3.37^, H137^4.56^, R183^5.39^ and L186^5.42^ has a larger value of 2–4 Å. In the homology models, Y91^3.37^ is more pointed toward helices 4 and 5, whereas it faces helix 6 forming interactions with Y240^6.51^ in the crystal structure. We noted that helix 3 in FFA1 has a proline kink at position 3.35 causing a slightly outward position of the helix in the crystal structure, which appears to create space for Y91^3.37^ to orientate toward and interact with Y240^6.51^ (Additional file 1: Figure 1S).

H137^4.56^ is pointed inside the helical bundle in the rhodopsin and PAR_1_ -based models, whereas it is more outside in the crystal structure. As noted by Srivastava *et al*. [16] helix 4 in FFA1 has glycine at position 4.58 and does not contain the frequently conserved proline at position 4.59, providing some flexibility to the upper part of helix 4, and thus explaining the different position of H137^4.56^.

R183^5.39^ and L186^5.42^ are similarly oriented inside the helical bundle in the modelled and crystal structures but there is a 4 Å shift in the backbone position of these residues, the result of a more inward position of helix 5 in the FFA1 crystal structure (Fig. 2). This brings R183^5.39^ closer to R258^7.35^ allowing charge pairing of guanidium side chains. None of built homology models had these interactions.

The network of interactions involving two arginines and two tyrosines in the FFA1 crystal structure closes the access to the deeper cavity at FFA1, whereas the absence of this network of interactions in the homology models provides a large cavity between helices 3, 4 and 5 (Additional file 1: Figure 1S). This difference has a major impact on ligand orientation in the modelled and crystal structures.

**Fig. 3:**
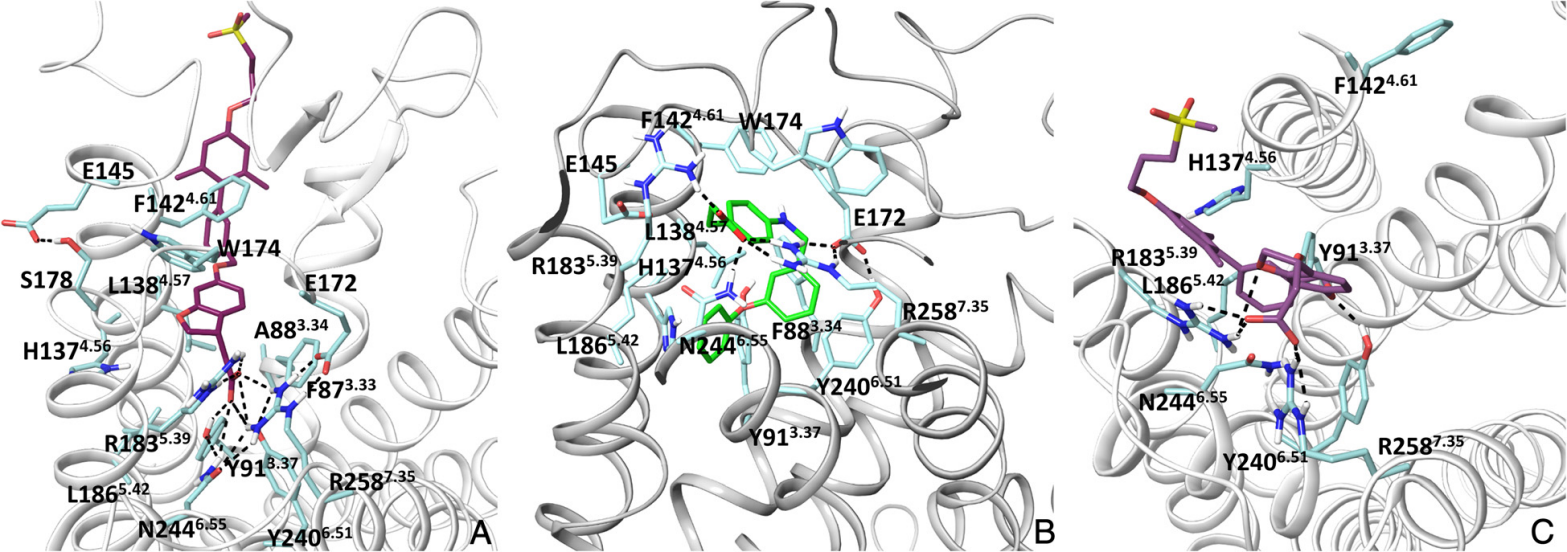
The ligand binding mode at the FFA1 crystal structure and homology models. **a**: the binding model of TAK-875, the ago-allosteric ligand in the FFA1 crystal structure, the ligand is pointed between helices 3 and 4 (mode 1 in the text). **b**: The binding mode of GW9508, the high potency agonist in the previous rhodopsin-based homology model of FFA1. The model was obtained from ref 12. **c**: The binding mode of TAK-875 in the PAR_1_-based model of FFA1. Hydrogen bonds are in black

In the crystal structure the carboxyl group of TAK-875 forms direct interactions with R183^5.39^, R258^7.35^, Y91^3.37^ and Y240^6.51^ through H-bonding [16] (Fig. 3a). Although homology modelling in conjunction with mutagenesis predicted direct interactions with these residues for linoleic acid, GW9508 and TAK-875, interactions with Y91^3.37^ and Y240^6.51^ were predicted through aromatic and hydrophobic contacts (Fig. 3b) [13, 14]. The deep cavity inside the helical bundle (Additional file 1: Figure 1S) in the earlier homology models locks agonists within the helical bundle allowing the hydrophobic tail of the ligand to interact with the tyrosines. In this position, GW9508 was predicted to form aromatic and amino-aromatic interactions with H137^4.56^ and hydrophobic interactions with L186^5.42^ (Fig. 3b). These interactions are not observed with TAK-875. H137^4.56^ and L186^5.42^ are at the distance of >6 Å from TAK-875 in the crystal structure. Instead, the hydrophobic part of TAK-875 interacts with F87^3.33^, F142^4.61^, W174^EL2^ and L138^4.57^ and is pointed between helices 3 and 4 facing lipids in the experimental structure (Fig. 3a). This docking position could not have been predicted in the homology models as the space between helices 3 and 4 is 2 Å narrower and residues F87^3.33^ and L138^4.57^ create an obstacle to the interhelical space. In contrast, docking is to some extent biased to the cavity formed by helices 4, 5 and 6 as the hydrophobic moiety of ligands in the crystal structures of rhodopsin and the β_2_ receptor is in this location. Docking to the FFA1 homology model based on the PAR_1_ receptor crystal structure predicts a different position for the hydrophobic part of an agonist compared to the rhodopsin- and β_2_ -based templates. Thus, the hydrophobic tail of the ligand is between helices 4 and 5 with the possibility of interacting with lipids (Fig. 3c). This orientation is also similar to the position of the ligand in the PAR_1_ receptor crystal structure. Clearly, the choice of a template for homology modelling is critical for establishing the size and shape of the binding cavity which, in turn, influences ligand docking solutions.

The second extracellular loop (EL2) was challenging to model due to the absence of any sequence similarity with the available templates, though modelling has predicted H-bonding between E172^EL2^ and R258^7.35^ correctly (Fig. 3a) [17]. E145^EL2^ has been anticipated to be important in receptor activation by interacting with R183^5.39^ in the homology models [17] but has H-bonding with S178^EL2^ in the crystal structure (Fig. 3a).

In the crystal structure no water molecules are present in the TAK-875 binding cavity, however there are water molecules that are involved in the H-bond network of interactions with the residues of the FFA1 binding cavity. A water molecule (HOH2533) forms a water-mediated hydrogen bond network of interactions with N244^6.55^, R258^7.53^ and S243^6.54^ (Additional file 2: Figure 2S). The residue N244^6.55^ predicted to be important for agonist binding through hydrogen bonding in the earlier models contributes to agonist binding indirectly via coordinating anchoring arginines. In addition, several water molecules connect EL2 and helices 5 and 7 via interactions with W174 ^EL2^, E172 ^EL2^, R183^5.39^ and R258^7.53^ (Figure 2S). The site map search predicts the binding cavity within this area (Additional file 3: Figure 3S).

We noted that K62^2.60^ faces the binding cavity in the FFA1 crystal structure (Fig. 2), similar to the corresponding residue in the peptide (PAR_1_, opioid and CXCR_4_), lysosphingolipid (S1P_1_) and nucleotide (P2Y_12_) receptor crystal structures, whereas it is at front of the lipid side in the earlier homology models. The location of a residue at position 2.60 in the binding site is due to proline at position 2.58 that causes the kink altering the packing of neighbouring residues. The role of K62^2.60^, an additional positively charged residue in the binding cavity of FFA1 is unknown and mutation of this residue could be of a great interest to explore its role in ligand binding. It is tempting to suggest that K62^2.60^ could be involved in interacting with allosteric modulators, thus the site map search suggests the binding cavity near K62^2.60^ (Additional file 3: Figure 3S).

In summary, the low-resolution homology models were helpful in establishing the location of the ligand binding site within helices 3, 5, 6 and 7 and the key residues for ligand anchoring. However, homology models were not accurate in predicting the nature of all ligand-receptor contacts and the precise ligand-binding mode. It appears that the position of helices 3, 4 and 5 affects docking solutions. It should be noted that the crystal structure of FFA1 is only available in the complex with the ago-allosteric ligand, which is assumed to bind in the overlapping region of the allosteric and orthosteric binding sites, thus binding of orthosteric agonists could be distinct from TAK-875 binding.

#### Ligand docking

**Fig. 4:**
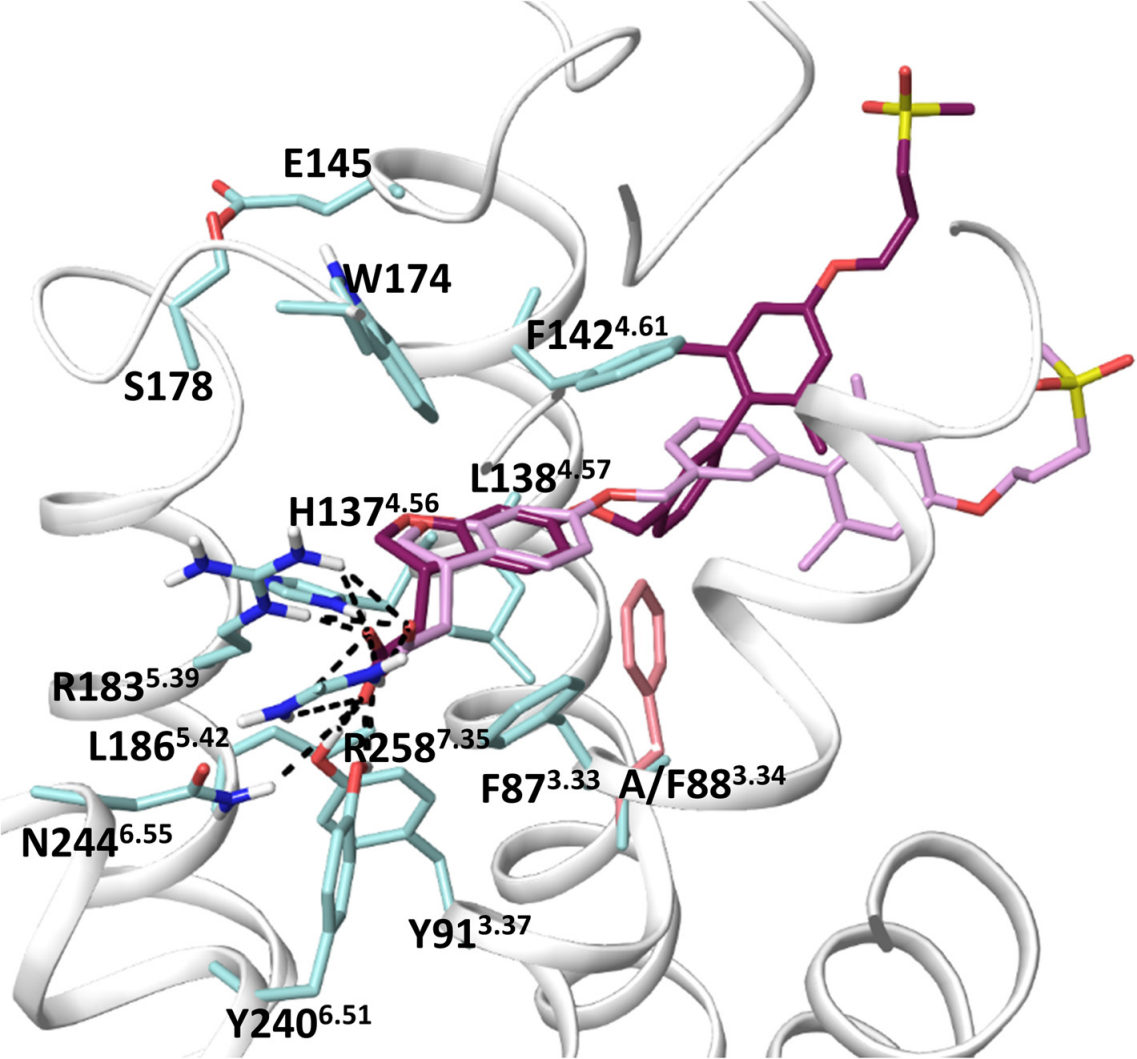
The superimposition of the FFA1 crystal structure and the homology model of the FFA1 wild type bound to TAK-875. The FFA1 wild type model was constructed from the FFA1 crystal structure using Prime [43]. TAK-875 was docked to the wild type model using Glide [20]. Phenylalanine mutation at position 3.34 is shown in pink

Because the crystal structure of FFA1 is available only in a complex with TAK-875 we next explore docking of a linoleic acid and synthetic agonists in the experimental FFA1 structure. Initially, we validate a docking protocol of the Glide program [20] by re-docking TAK-875 to the crystal structure to examine whether it can reproduce the experimental binding pose of TAK-875. In all generated poses, the Glide standard docking protocol [20] has reproduced the position of TAK-875 close to the crystal structure position with a RMSD value of 0.8 Å. Notably, alanine at position 3.34 in the crystal structure (Fig. 3a) is a mutation being used together with L42A, G103A and Y202F to thermostabilise the protein [16]. The alanine is located ~5 Å from TAK-875, whereas phenylalanine at this position in the wild type could be at distance of 2 Å. Given its potential impact on a ligand position we rebuilt the wild type of FFA1 and docked TAK-875 (Fig. 4).

**Fig. 5:**
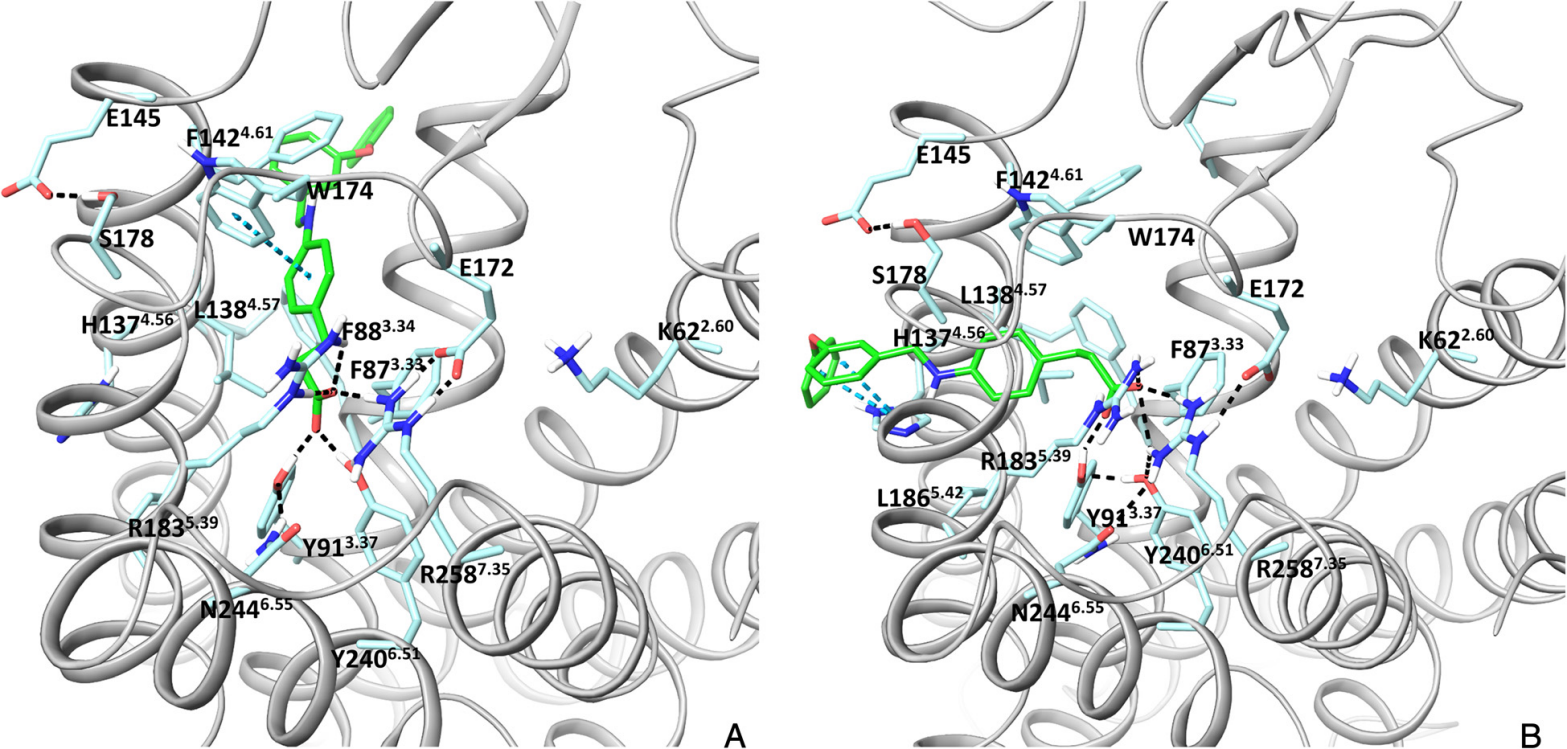
Two proposed binding modes for the GW9508 agonist in FFA1. **a**: mode 1, similar to the binding of TAK-875 in the FFA1 crystal structure. **b**: mode 2, the alternative pose derived from flexible docking. Hydrogen bonds, π- π and π-cation interactions are in black and blue, respectively. The binding site residues, E145 and E172 of the second extracellular loop predicted to form salt bridges in the earlier models and K62^2.60^ predicted here to form the allosteric binding site are shown

The ligand is docked in the similar orientation but with the higher RMSD of 3 Å, mainly for the biphenyl tail. To achieve this result, the docking protocol with van der Waals radius of protein atoms soften from 1 to 0.8 Å was used. We anticipate that mutation at position 3.34 in the crystal structure could correct the agonist position. It appears this change however is not dramatic for the TAK-875 binding affinity [16]. Linoleic acid and GW9508 were placed similar to TAK-875 in the binding site. The docking position of GW9508, as an example is shown in Fig. 5a.

To account for flexibility of side chains in the binding site we use the InducedFit docking protocol [21], where initial ligand docking is followed by side-chain sampling and final re-docking into newly-generated receptor conformations. Interestingly, we found that TAK-875, linoleic acid and GW9508 have two binding modes in one of which the hydrophobic tail of the agonists, similar to the co-crystalized TAK-875, is pointed in the space between helices 3 and 4 (Fig. 5a) (mode 1), whereas in another one the hydrophobic tail of the agonists is pointed in the space between helices 4 and 5 (mode 2), Fig. 5b. Short simulations of the agonist-receptor complexes show that the ligands maintain stably the binding mode obtained in docking studies. In both modes of binding, the carboxyl group of ligands forms H-bonding with two arginines and two tyrosines. In mode 2, GW9508 is able to form aromatic and hydrophobic interactions with H137^4.56^ and L186^5.42^ predicted to be important in the earlier homology models. Thus, Phe and Ala mutations of H137^4.56^ reduce the potency of GW9508 by 28- and 100-fold, respectively [13, 14]. Phe mutation of L186^5.42^ lowers the potency of GW9508 by 30-fold [14]. Our docking experiments show that this binding mode dominates in the FFA1 wild type.

To further explore the induced fit, we conducted conformational search of the FFA1 binding site residues of the crystal structure without a ligand and found the side chain of F87^3.33^, F142^4.61^, L138^4.57^ and W174^EL2^ residues closing the interhelical space of helices 3 and 4 in many generated conformations (Additional file 4: Figure 4S). Notably, Glide and InducedFit docking to these receptor conformations gives mode 2 as a favourable solution. Based on our flexible docking experiments we hypothesize the possibility of an alternative binding mode for orthosteric agonists.

Next, we explored Glide and InducedFit docking of other agonists - TUG-770 [22], AMG837, AM1638 and AM8182 [23] of FFA1. TUG-770 is a highly potent agonist demonstrating profound effects on glucose tolerance in mice [22]. AMG837 is a partial agonist, whereas AM1638 and AM8182 are full agonists; all three appear to elicit activation through distinct binding sites [23, 24].

Our docking results show different docking preferences. Mode 1 in Glide docking could be obtained with low energy only for AMG837 (Additional file 5: Figure 5S). The InducedFit docking predicts both modes of binding with some preference toward mode 2 for AM8182 and TUG-770 (Additional file 5: Figure 5S). The different preference in docking modes could provide some structural insight into a distinct response of AMG837 and AM8182 to mutation of arginines at positions 5.39 and 7.35 in pharmacological studies [23]. Mode 2 for TUG-770 could be helpful in interpretation of the SAR studies, indicating the preference of *meta*- over *ortho*- position of a substituent in the terminal ring. This is because in this mode the ligand with the *meta*-group has an extra interaction with H137^4.56^ (Additional file 5: Figure 5S). The bulky AM1638 compound (Fig. 1) could not be docked in the specified docking site involving two arginines and two tyrosines. Indeed AM1638 is unaffected by arginine mutations [23] in pharmacological assays. We hypothesise here that AM1638 could bind to the binding site of the receptor involving K62^2.60^.

To further validate the ligand binding modes at FFA1, mutagenesis of the residues lining the interhelical space of helices 3 and 4 as well as helices 4 and 5 will be beneficial. Thus, the receptor with single or multiple mutations could be constructed, where the replacement is made to bulky residues or residues with opposite physicochemical property to prevent binding to one of the regions. Analysis of such receptor mutants on a panel of agonists with similar and different chemical scaffolds as well as pharmacological properties (full agonists, partial agonists, ago-allosteric agonists and biased agonists) may decipher precise binding modes of agonists chemically distinct from the co-crystallized TAK-875.

From docking to the FFA1 crystal structure we predict the alternative ligand binding mode at the interhelical space between helices 4 and 5, which could accommodate the orthosteric agonists. This binding mode can explain mutagenesis results for H137^4.56^ and L186^5.42^ [13].

### Free fatty acid receptors 2 and 3

#### Structural models

**Fig. 6:**
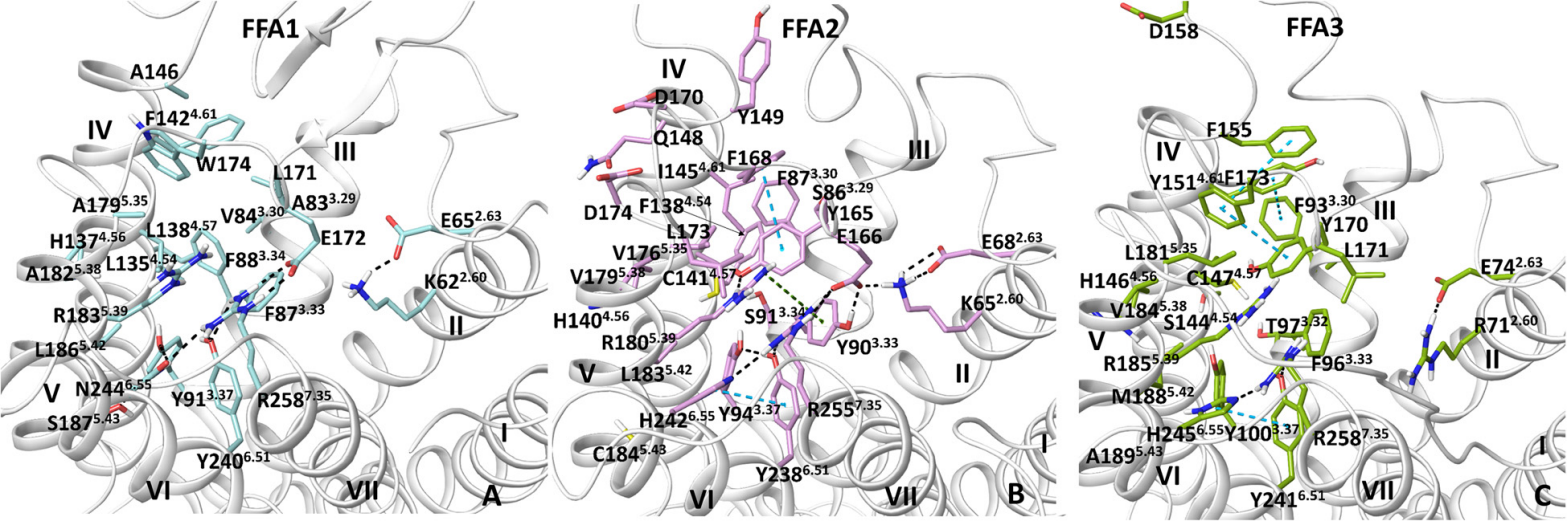
The ligand binding site of the free fatty acid 1–3 receptors **a**: the binding site in the FFA1 crystal structure; **b** and **c**: the binding site in the FFA1-based homology model of FFA2 and FFA3, respectively. The side chain of the binding site residues in FFAs is shown in blue, pink and green, respectively. H-bonding, π- π and π-cation interactions are shown in black, blue and green dotted lines, respectively

The sequence identity of the FFA2 and FFA3 helical bundle with FFA1 is 32 % and 33 %, respectively, which is less than the sequence identity between the subtypes of the adrenergic, muscarinic and opioid receptors (>60 %), suggesting more structural differences between the FFA receptor subtypes. The sequence identity is higher between FFA2 and FFA3, 49 %. Sequence alignment of the FFA receptors is in Figure 6S (Additional file 6).

**Fig. 7:**
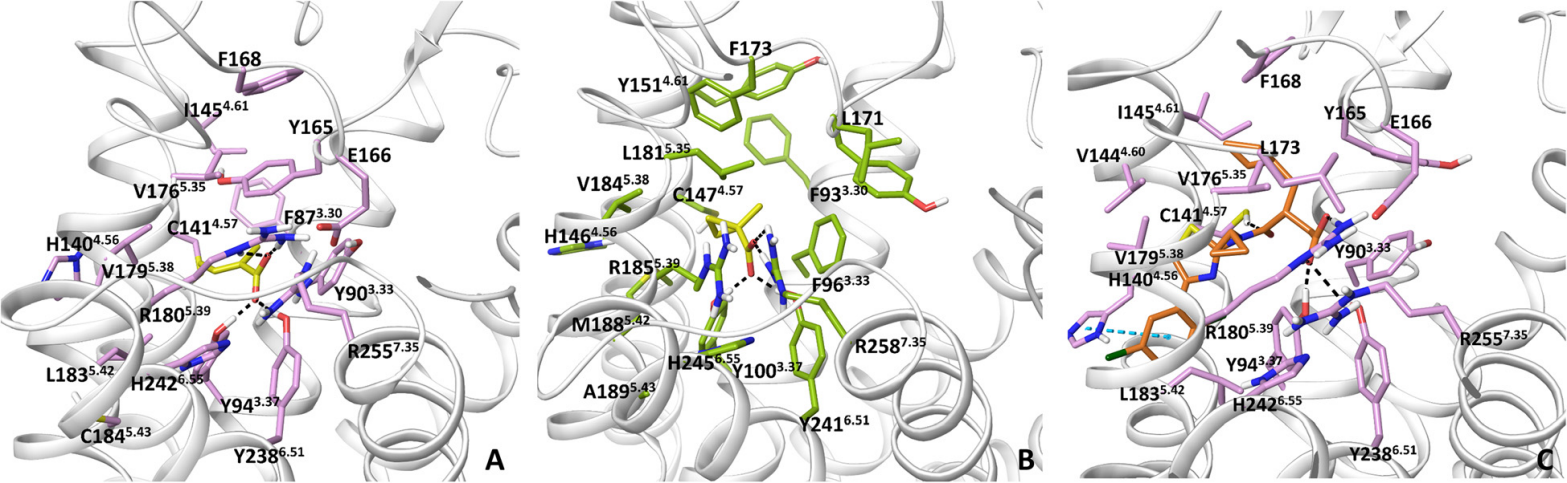
Ligand binding in FFA2 and FFA3. **a**: the binding mode of tiglic acid at FFA2. **b**: the binding mode of 1-MCPC in FFA3. **c**: the binding mode of **1** in FFA2. FFA2-3 homology models are based on the FFA1 crystal structure. H-bonding and π- π interactions are shown in black and blue dotted lines, respectively

Among other currently available GPCR templates for homology modeling (Additional files 7 and 8: Figure 7S and Table 1S), the highest sequence identity of FFA2 and FFA3 within the transmembrane helices is with the purinergic P2Y_12_ receptor for FFA2 (30 %) and the PAR_1_ receptor for FFA3 (33 %). The first models of FFA2 and FFA3 to study the ligand-receptor interactions were constructed using the β_2_ receptor crystal structure with the sequence identity of 17 % [10, 25]. We here evaluate the FFA1-based template in modeling of the FFA2 and FFA3 binding site and ligand-receptor interactions.

Examination of the sequence alignment shows that unlike FFA1 helices 3 and 4 in FFA2 and FFA3 don’t have proline and glycine in the upper side, indicating possible variation of the helix position from FFA1.

Similar to FFA1, residue mutagenesis at FFA2 and FFA3 shows that the conserved arginines at positions 5.39 and 7.35 and histidine at position 6.55 are important for coordinating the binding of short chain fatty acids at FFA2 and FFA3 [26]. Unlike other 25 available templates for homology modelling (Additional file 7: Figure 7S), the FFA1 template provides a favorable starting position of the lengthy arginines in the FFA2 and FFA3 homology models for ligand docking. Manual side chain adjustments to bring the arginine residues together for the interaction with the carboxyl group of a ligand are needed when using other templates. Figure 8S (Additional file 9) shows the superimposition of FFA2 homology models built using 25 GPCRs with available crystal structures, with the visualized arginine residues to appreciate various positions of the arginine side chain. It is therefore expected that the FFA1-based homology models will be more suitable for characterizing the FFA2 and FFA3 binding site compared to the P2Y_12_ and PAR_1_ –based homology models.

Comparison of FFA2 with FFA1 structure reveals a more intensive network of interactions within the FFA2 binding site due to extra hydrophilic and aromatic residues (Fig. 6, a and b). Thus, Y165^EL2^ in FFA2 (L171 in FFA1) and Y90^3.33^ (F87 in FFA1) contribute to aromatic and H-bond networks of interactions with conserved Y94^3.37^, R180^5.39^, Y238^6.51^ and R255^7.35^. Residue H242^6.55^ (N244 in FFA1) forms an H-bond with R255^7.53^ and aromatic and hydrophobic interactions with Y94^3.37^ and Y238^6.51^. In addition, residues K65^2.60^, E68^2.63^ and E166^EL2^ contribute to an H-bonding network within the binding site. The major difference with the previous FFA2 homology models [10, 25] is that we didn’t observe this intensive network of hydrogen bond and aromatic interactions. The higher quality FFA2 homology model suggests that the binding site is more packed and less opened from the extracellular side.

The interhelical space between helices 3 and 4 is less accessible in FFA2 compared to FFA1 as it is filled by residues F87^3.30^ (V84 in FFA1), I145^4.61^ (F142 in FFA1) and F138^4.54^ (L135 in FFA1). In addition, S91^3.34^ (F88 in FFA1) and C141^4.57^ (L138 in FFA1) are in very close proximity to form an H-bond, further closing the hole between helices. Non-conserved residues such as V176^5.35^ (A179 in FFA1), V179^5.38^ (A182 in FFA1) and F168^EL2^ (W174 in FFA1) also change the entry to the interhelical space between helices 3 and 4 in FFA2. Overall, the binding cavity is smaller in FFA2 (332 Å^3^) than in FFA1 (400 Å^3^) (Table 1), providing a geometrical reason to a preference in binding of short (≤C3) over medium (≥C6) and long chain (≥10) FFAs.

**Table 1:** Properties of the binding cavity formed by helices 3–7

Receptor	Volume Å^3^	SASA Å^2^	SASA_phil_ Å^2^	SASA_phob_ Å^2^	SASA_aliph_ Å^2^	SASA_arom_ Å^2^
FFA1	400	690	138	598	511	87
FFA2	332	451	208	465	309	159
FFA3	385	455	285	426	320	96

SASA is the solvent accessible surface area of the residues forming the binding cavity, SASA with subscript: *phil*, *phob*, *aliph* and *arom* is SASA of the hydrophilic, hydrophobic, aliphatic or aromatic residues, respectively

Similar to FFA2, the interhelical space between helices 3 and 4 is less opened in FFA3 due to non-conserved residues, F93^3.30^ and Y151^4.61^, filling the interhelical space (Fig. 6). Compared to FFA1 and FFA2 the H-bonding network at FFA3 including residues at positions 3.37, 5.39, 6.51 and 7.53 has a different arrangement likely as a result of the non-conserved residues L171^EL2^ (GLU in FFA1/FFA2) and PHE at position 3.33 (TYR in FFA2) that are unable to form H-bonding. Overall, smaller number of H-bonds is observed in FFA3 compared to FFA2. The binding cavity in FFA3 is larger (385 Å^3^) compared to FFA2 but smaller than in FFA1 (Table 1). The new models suggest that Y165^EL2^ in FFA2 and Y170^EL2^ in FFA3, which corresponds to L171 ^EL2^ in FFA1 are located in the center of the FFA orthosteric site and contributes to reduction of the volume in FFA2 and FFA3, playing likely an important role in subtype selectivity between FFA1 and FFA2-3.

We characterize the physicochemical properties of the ligand binding sites at FFAs by calculating the solvent accessible surface area (SASA) of the hydrophilic, hydrophobic, aromatic and aliphatic residues of the binding cavity at FFAs (Table 1). As expected, the SASA of the hydrophobic residues is significantly higher compared to the SASA of the hydrophilic residues in all the receptors. The higher percent of hydrophilicity is in FFA3 (43 %) compared to FFA2 and FFA1 (30 and 19 %, respectively). The SASA of aromatic residues has the higher value for FFA2, 159 Å^2^, whereas it is 87 and 96 Å^2^ in FFA1 and FFA3, respectively. The SASA of aliphatic residues has the higher value for FFA1, 511 Å^2^ compared to 309 and 320 Å^2^ in FFA2 and FFA3, respectively. It appears that several distinct binding regions within the binding cavities of FFAs could be mapped that were not so clear in the earlier models.

The EL2 of FFA2 and FFA3 is six- residues shorter than the EL2 of FFA1. There is some similarity in the EL2 sequence after the disulfide bridged cysteine. The EL2 sequence identity is higher between FFA2 and FFA3 (Additional file 6: Figure 6S). Compared to other available templates, the EL2 of FFA1 is the most suitable template for EL2 modeling of FFA2 and FFA3.

Residues L173^EL2^, Y165^EL2^ and E166^EL2^ of FFA2 (Fig. 6b) known to be important for agonist binding from mutagenesis [25] face the binding cavity in the new FFA2 model and likely contribute to agonist binding through a network of interactions with a ligand or residues of the helices. The residue Q148^EL2^ (Fig. 6b) gives substantial loss in the potency of **1** (Fig. 1) in FFA2 when it is mutated to glutamate and contributes to ligand selectivity in the pharmacological studies [25]. The new FFA2 model suggests that Q148^EL2^ is situated outside of the binding site and contributes to ligand binding likely indirectly through potential H-bonding with nearby D170^EL2^ or D174^EL2^ (Fig. 6b). The glutamate substitution of Q148^EL2^ [25] might therefore lead to repulsion and subsequent change in the EL2 conformation, which can affect the ligand binding. D170^EL2^ and D174^EL2^ are non-conserved in FFAs and could be of interest for mutagenesis. The conformation of EL2 in FFA2 and FFA3 cannot suggest a possible ionic salt bridge of G159E^EL2^ mutation in FFA2 or D158^EL2^ in FFA3 (Fig. 6c) with one of the arginines of the binding site, holding the receptor in the inactive state, as it was proposed for interpretation of the reduced constitutive activity of the receptors in functional essays [27]. These residues are far away from arginines in the new structural models of the receptors. It should be noted that the low similarity of the EL2 sequence in this region with FFA1 does not allow to model a precise position and interactions of Q148^EL2^ in FFA2 and G159 ^EL2^ in FFA3.

Overall, the new homology models reveal the role of non-conserved residues in defining a distinct network of interactions in the ligand binding cavity of FFAs, which appears to be influential for FFA subtype selectivity.

### Ligand docking

Docking of the selective short carboxylic acids [10], FFA2-selective tiglic acid and FFA3-selective 1-MCPC to the FFA1-based model of FFA2 and FFA3 indicates that the carboxyl group of the fatty acids is anchored by conserved R180^5.39^, R255^7.35^, Y94^3.37^ and Y238^6.51^. The non-conserved residues at positions 5.43 and E166/L171^EL2^ predicted to be important for FFA2/FFA3 selectivity together with the residue at position 5.42 in the earlier homology models contribute more to the shape of the binding cavity rather than direct interactions with a ligand (Fig. 7a and b). Instead, the non-conserved residue at position 5.42 is at distance of 4 Å enabling it to form van der Waals interactions. The hydrophobic part of the tiglic acid forms hydrophobic contacts with Y90^3.33^, Y94^3.37^, C141^4.57^, I145^4.61^, V179^5.38^ and Y165^EL2^ in FFA2, whereas 1-MCPC interacts with F96^3.33^, L181^5.35^, V184^5.38^, R185^5.39^, M188^5.42^ and F173^EL2^ in FFA3. The new structural models suggest a small binding cavity in FFA2 due to bulky aromatic residues and an intensive H-bonding network, structurally explaining a preference in binding of carboxylic acids with sp2 hybridized α-carbons to FFA2, whereas a larger binding cavity in FFA3 with a lesser network of interactions could be a reason for a preference in binding of carboxylic acids with sp3 hybridized α-carbons to FFA3 as observed in pharmacological studies [10]. We predict that the residues that determine subtype selectivity between FFA2 and FFA3 are Y90^3.33^, I145^4.61^ and E166^EL2^ in FFA2 and the corresponded F96^3.33^, Y151^4.61^ and L171^EL2^ in FFA3.

In addition, we have docked **1**, a highly potent synthetic agonist to the FFA1-based model of FFA2 to compare with the earlier ligand binding mode predicted from the β_2_-receptor-based homology model and mutagenesis [25]. Both Glide and InducedFit protocols dock **1** to the new FFA2 model in a similar fashion. In particular, the carboxyl group of the ligand contributes to a network of interactions through H-bonding involving Y90^3.33^, Y94^3.37^, R180^5.39^, Y238^6.51^, H242^6.55^ and R255^7.35^ (Fig. 7c), similar to TAK-875 binding in the crystal structure of FFA1 and different from earlier predictions, where tyrosines had hydrophobic interactions with **1**. The aromatic moiety of the agonist is pointed between helices 4 and 5 forming aromatic interactions with H140^4.56^ and hydrophobic contacts with V179^5.38^ and L183^5.42^. Similar to FFA1, histidine at position 4.56 has some notable effect on the activity of fatty acids in FFA2 and FFA3 [26, 28] and greatly reduces the activity of 1, a highly potent synthetic agonist in FFA2 [25]. This orientation is different from the position of the TAK-875 hydrophobic tail but similar to predicted agonist binding mode 2 in FFA1. The cyclopropyl group of **1** forms hydrophobic interactions with V176^5.35^, V179^5.38^ and R180^5.39^, and the cyclopentyl group makes contacts with C141^4.57^, V144^4.60^, I145^4.61^ and F168^EL2^. The importance of residues at positions 3.33, 4.56, 5.38, 5.39, 6.51, 6.55 and 7.35 in binding of **1** is supported by mutagenesis [25]. The new model suggests the residues at positions 3.37, 4.60, 4.57, 5.35 and F168^EL2^ are now involved in direct interactions with **1**.

Allosteric agonists, AMG7703 [12, 29] of FFA2 and **2** of FFA3 [30] (Fig. 1) have been recently identified. Alanine mutation of arginines at positions 5.39 and 7.35 does not affect the binding of these ligands in the corresponding receptors, indicating that a binding site of these ligands is distinct from free fatty acids [30, 31]. Several residues of the FFA2 receptor extracellular cavity predicted based on the β2 homology model have been mutated to identify the binding site of AMG7703 but none of the mutations substantially affected the potency of AMG7703 [28, 31]. It has been only shown experimentally that the allosteric property of AMG7703 is prevented by the L173A^EL2^ mutant or substitution of EL2 with that of FFA3 [31]. In the new FFA2 model, L173^EL2^ faces the binding cavity and links the EL2 loop with helix 6, potentially involved in the ligand binding process and subsequent conformational changes leading to activation. Similar to FFA1 allosteric ligands, we hypothesize that the extracellular side involving helices 1, 2 and 7 with the positively charged residue at position 2.60 (LYS in FFA2 and ARG in FFA3) could form the allosteric binding site in FFA2 and FFA3 (Additional file 3: Figure 3S).

In summary, the novel FFA2 and FFA3 models further delineate the orthosteric agonist binding site by interpreting the few available mutagenesis data. Importantly, the novel models highlight non-conserved residues lining the orthosteric binding site at FFA2 and FFA3 that could be used for further mutagenesis studies. Interestingly, unlike FFA1, the FFA2 agonists cannot be easily docked to the interhelical space between helices 3 and 4, however we cannot exclude this option. Similar to FFA1, mutagenesis of residues in helices 3, 4 and 5 and binding studies with different agonists would help in establishing ligand binding modes at FFA2. The higher quality homology models could be now probed in *in silico* structure-based design, helping to discover the first FFA3 orthosteric ligands. Finally, novel models suggest the location of the allosteric agonist binding site.

## Conclusions

The free fatty acid receptors are recently discovered G protein-coupled receptors with potential impact to improve the life of patients suffering from diabetes, obesity and immune diseases. However, the absence of a large pool of various small molecule modulators with high potency and selectivity as well as acceptable pharmacokinetic properties create an obstacle to characterize FFA biology and physiology and subsequent validate these drug targets in the clinics. The published crystal structure of FFA1 in the complex with TAK-875 in the last year [16] represents the first direct structural knowledge of binding at the FFA1 receptor and facilitates the exploration of the binding sites at FFA2 and FFA3.

Our comparison of the previous models and the crystal structure of the FFA1 receptor shows that the homology models based on templates with 16–18 % sequence identity were useful in locating the binding site at FFAs and identifying key residues for binding but not accurate in establishing the precise network of ligand-protein interactions and the ligand binding mode. Furthermore, modelling of EL2 is a challenging task to complete and this challenge still remains for FFA2 and FFA3 as there is low sequence similarity with FFA1. Our findings are in the line with conclusions made from community-based assessments of GPCR modelling on the example of the adenosine A_2A_, dopamine D_3_, chemokine CXCR4, serotonin 5HT_1B_ and 5HT_2B_ and smoothened receptors [32–34].

From modelling of the FFA1 receptor we posit that when the sequence identity is low (<20 %) with respect to the receptors with available crystal structures, it is useful to explore several templates for homology modelling. Thus, crystal structures of templates with differing positions of a ligand in the binding cavity can help to examine various ligand binding modes in docking studies. Care should be taken with helices containing proline and glycine as these residues can remarkably change side chain packing and vary the position of the extracellular tips of the helices creating a distinct shape and size of the binding cavity, thus affecting docking conditions. Furthermore, multiple templates could help in exploring various orientations of the side chain of lengthy residues within the binding site. Thus, one of the challenge for FFA modelling was to predict a favourable position of the arginines for ligand docking. The importance of multiple templates for GPCR modelling has been discussed in literature using other receptors as a case study [35, 36].

Docking to the FFA1 crystal structure predicts an alternative binding mode for agonists, in which the ligand hydrophobic tail is at the interhelical space between helices 4 and 5. A similar mode of binding is predicted for FFA2 agonists. This binding mode can be linked with the available mutagenesis data. The FFA1-based models of FFA2 and FFA3 suggest hydrogen bonding of the agonist carboxyl group with two tyrosines at positions 3.37 and 6.51, in addition to two anchoring arginines. Overall, the new structural models of FFAs give more details on molecular reasons for the preference in binding of long or short chain fatty acids by highlighting the difference in the network of interactions between the receptors and identifying the position of non-conserved residues, which contribute to the shape and volume of the binding sites. In particular, we predict residues at positions 3.33, 4.61, 6.55 and two residues followed the disulfide bridged cysteine in the second extracellular loop play a central role in FFA subtype selectivity.

Novel structural models of FFAs suggest the location of an allosteric binding site. Thus, the cavity between helices 2 and 7 and EL2 with a putatively anchoring role of the residue at position 2.60 is predicted. This residue has not been appreciated in earlier homology models as it was pointed outside of the helical bundle. To validate the importance of this residue in binding of allosteric modulators will require mutagenesis experiments. Interestingly, this positively-charged residue in helix 2 makes the FFAs binding cavity somewhat similar with the FFA4 (GPR120) receptor – the receptor with a preference in binding of the long chain fatty acids, which has the positive-charged residue at position 2.64 coordinating the carboxyl group of agonists [37, 38]. Despite binding the long chain fatty acids like FFA1, this receptor has substantially low sequence identity with FFAs (<19 %).

FFA structural models provide more details on receptor-agonist interactions and suggest novel predictions for mutagenesis and medicinal chemistry. Further insights in ligand binding modes at FFAs will be fuelled by availability of receptor mutagenesis data for various ligands, including orthosteric and allosteric agonists and antagonists in the short term and validated by new crystal structures of FFAs bound to different ligands in the long term.

## Methods

### Homology modelling

The FFA1 crystal structure with the PDB code of 4PHU was used for ligand docking and homology modelling of FFA2-3. Homology models were also built based on the crystal structures with PDB code of 1U19, 3CAP, 3PBL, 3RZE, 2RH1, 4AMJ, 3V2Y, 4EIY, 3ODU, 4DAJ, 3UON, 4DJH, 4DKL, 4EJ4, 4EA3, 3VW7, 4GRV, 4IAR, 4IB4, 4MBS and 4NTJ to compare with the FFA1 crystal structure and produce Figure 8S. The sequence alignment strategy for the GPCR family is described by *Costanzi* [39] is used in this work. Briefly, multiple sequence alignment was built using CLUSTALW [40] with the BLOSUM62 matrix [41] for helices. Subsequent visual inspection of the alignment and manual adjustment was performed to match the helix conserved motifs of GPCRs: GX_3_N for helix 1, N(S,H)LX_3_DX_7,8,9_P for helix 2, SX_3_LX_2_IX_2_D(E,H)RY for helix 3, WX_8,9_P for helix 4, FX_2_PX_7_Y for helix 5, FX_2_CW(Y,F)XP for helix 6 and LX_3_NX_3_N(D)PX_2_YX_5,6_F for helices 7 and 8, where X_n_ is *n* neighbouring non-conserved residues and residues in parentheses substitute the preceding residue. Due to the low sequence similarity and length variability of the loops, the alignment is performed in a pairwise manner comparing the target receptor with a template receptor. In the case of the FFAs family, all the three members of the family were aligned for the loop alignment (Additional file 6: Figure 6S). The sequence alignment between FFA1-3 and 25 GPCRs with the available crystal structures (Additional file 7: Figure 7S) was also verified using the HHpred server [42], which uses the pairwise comparison of profile hidden Markov models. This server has predicted correctly all the transmembrane helices and identified the conserved motifs. Unlike the BLOSUM-based alignment no manual refinements are need to be made for loop regions. The probability, E-values and p-values (Additional file 7: Table 2S) from HHpred server show high reliability of the alignment. The homology models were generated with Prime 3.8 [43] using the default settings. The models were refined using a default energy minimization protocol implemented in Prime 3.8.

### Molecular docking

The FFA1 crystal structure was prepared with the protein preparation utility of Maestro 9.9 [44]. Molecular docking to FFA1-3 was conducted using Glide 6.5 [45]. The receptor grid was defined by selecting residues at positions 3.37, 4.57, 5.39, 6.51 and 7.35. The standard precision (XP) Glide protocol without any constraints was used for docking. The InducedFit protocol [20, 21, 43, 45, 46] with the docking box defined using the residues above was used and no side chain trimming was applied in a re-docking part of the protocol. For FFA2, the side chain trimming was applied for Y165 and Y149 that widely occluded the binding cavity. The best scored binding pose of TAK-875 was compared to the co-crystallized orientation of TAK-875 and the RMSD was calculated as a measure of docking reliability. For the identified ligand binding modes, the best docking pose was chosen based on the docking energy and agreement with residue mutagenesis data and ligand structure-activity relationship (SAR) data.

### Analysis of structural models

To investigate the water-mediated interactions, hydrogens are added and minimized using a standard protocol of the protein preparation utility of Maestro 9.9. For the SASA and volume calculations residues: P80, V81, A83, V84, F87, F88, L90, Y91, L135, L138, F142, V141, L158, A179, A182, R183, N244, Y240, R258, L171, W174, G143, G139 and E172 in FFA1 and the residues at the same positions in FFA2 and FFA3 were selected. The SASA and volume was calculated with VMD 1.9.2beta1 [47] and Chimera 1.6 [48], respectively. SiteMap 3.3 [49] with the minimal 9 site points was used to predict binding sites. The 500 ps molecular dynamics simulations were conducted to examine the stability of the ligand binding modes using MacroModel 9.9 [50]. OPLS_2005 force fields were used and the surface area-based version of the generalized Born model (GB/SA) were used for treatment of solvent and its dielectric constant was assigned a value of 1. Conformational search for FFA1 in the ligand-free form were performed using the Monte Carlo multiple minimum method as implemented in MacroModel 9.9 [50]. Residues located within 5 Å of GW9508 in modes 1 and 2 were subjected to extend torsional sampling. A shell of frozen atoms within 4 Å was also included in the conformational search, whereas the remaining atoms were excluded. 5000 steps of the Monte Carlo multiple minimum method were performed, and the resulting structures were saved with a potential energy lower than 100 kJ/mol. Figures of the molecular models were generated with Maestro 9.9 [44].

### Endnote

^a^ The number in superscript is the Ballesteros-Weinstein indexing system [51], where the most conserved residue in a given helix is assigned the index *X*.50 with *X* as the helix number, while the remaining residues are numbered relatively to position 50.

### Availability of supporting data

The figure of the binding cavities in the crystal structure and homology models, the figure of a H-bond network with water, the figure of putative allosteric sites, the figure of superimposition of the crystal structures with the representative structure from conformational search, the figure of the TUG-770, AMG837 and AM8182 binding mode, the sequence alignment of FFAs, the figure of superimposition of FFA2 homology models built based on 25 GPCRs and the table of helix sequence identity of FFAs and 22 GPCRs with available crystal structures. All the supporting data are included as additional files.

## Additional files

Additional file 1:
**The binding cavities in the FFA1 crystal structure (A), rhodopsin (B) and β2 adrenergic receptor (C) based FFA1 homology models.** (PDF 416 kb) Additional file 2:
**The hydrogen bonding network involving water molecules in FFA1.** (PDF 269 kb) Additional file 3:
**The location of putative allosteric sites at** **FFA1-3.** (PDF 310 kb) Additional file 4:
**The superimposition of the FFA1 crystal structure and a characteristic conformation of FFA1 from conformational search of the FFA1 crystal structure without the ligand.** (PDF 309 kb) Additional file 5:
**Agonist binding at the FFA1 crystal structure.** (PDF 415 kb) Additional file 6:
**Sequence alignment of the free fatty acid receptors.** (PDF 235 kb) Additional file 7:
**Sequence alignment of the free fatty acid receptors and 25 GPCRs with available crystal structures and HHpred server predictions of a homologous relationship.** (PDF 329 kb) Additional file 8:
**Sequence identity (%) in the transmembrane helices of the FFA receptors and 25 GPCRs with available crystal structures.** (PDF 343 kb) Additional file 9:
**Superimposition of 25 FFA2 homology models built based on 25 GPCRs with an available crystal structure.** (PDF 246 kb)

## Abbreviations

GPCRs: G protein-coupled receptors
FFAs: Free fatty acid receptors
FFA1: Free fatty acid receptor one
FFA2: Free fatty acid receptor two
FFA3: Free fatty acid receptor three
RMSD: Root-mean-square deviation
PAR1: Protease-activated receptor one
β_2_: β_2_ adrenergic receptor
CXCR_4_: Chemokine receptor
S1P_1_: Lysosphingolipid receptor
P2Y_12_: Nucleotide receptor
EL2: Second extracellular loop

## References

[1] Stoddart LA, Smith NJ, Milligan G (2008). International Union of Pharmacology. LXXI. Free fatty acid receptors FFA1, −2, and −3: pharmacology and pathophysiological functions. Pharmacol Rev.

[2] Briscoe CP, Peat AJ, McKeown SC, Corbett DF, Goetz AS, Littleton TR, McCoy DC, Kenakin TP, Andrews JL, Ammala C, Fornwald JA, Ignar DM, Jenkinson S (2006). Pharmacological regulation of insulin secretion in MIN6 cells through the fatty acid receptor GPR40: identification of agonist and antagonist small molecules. Br J Pharmacol.

[3] Itoh Y, Hinuma S (2005). GPR40, a free fatty acid receptor on pancreatic beta cells, regulates insulin secretion. Hepatol Res.

[4] Brown AJ, Goldsworthy SM, Barnes AA, Eilert MM, Tcheang L, Daniels D, Muir AI, Wigglesworth MJ, Kinghorn I, Fraser NJ, Pike NB, Strum JC, Steplewski KM, Murdock PR, Holder JC, Marshall FH, Szekeres PG, Wilson S, Ignar DM, Foord SM, Wise A, Dowell SJ (2003). The Orphan G protein-coupled receptors GPR41 and GPR43 are activated by propionate and other short chain carboxylic acids. J Biol Chem.

[5] Ulven T (2012). Short-chain free fatty acid receptors FFA2/GPR43 and FFA3/GPR41 as new potential therapeutic targets. Front Endocrinol (Lausanne).

[6] Dranse HJ, Kelly ME, Hudson BD (2013). Drugs or diet?--Developing novel therapeutic strategies targeting the free fatty acid family of GPCRs. Br J Pharmacol.

[7] Milligan G, Ulven T, Murdoch H, Hudson BD (2014). G-protein-coupled receptors for free fatty acids: nutritional and therapeutic targets. Br J Nutr.

[8] Bindels LB, Dewulf EM, Delzenne NM (2013). GPR43/FFA2: physiopathological relevance and therapeutic prospects. Trends Pharmacol Sci.

[9] Watterson KR, Hudson BD, Ulven T, Milligan G (2014). Treatment of type 2 diabetes by free Fatty Acid receptor agonists. Front Endocrinol (Lausanne).

[10] Schmidt J, Smith NJ, Christiansen E, Tikhonova IG, Grundmann M, Hudson BD, Ward RJ, Drewke C, Milligan G, Kostenis E, Ulven T (2011). Selective orthosteric free fatty acid receptor 2 (FFA2) agonists: identification of the structural and chemical requirements for selective activation of FFA2 versus FFA3. J Biol Chem.

[11] Hoveyda H, Brantis CE, Dutheuil G, Zoute L, Schils D, Bernard J: Compounds, pharmaceutical composition and methods for use in treating metabolic disorders. June 17, 2010, International patent application WO 2010/066682.

[12] Wang Y, Jiao X, Kayser F, Liu J, Wang Z, Wanska M, Greenberg J, Weiszmann J, Ge H, Tian H, Wong S, Schwandner R, Lee T, Li Y (2010). The first synthetic agonists of FFA2: Discovery and SAR of phenylacetamides as allosteric modulators. Bioorg Med Chem Lett.

[13] Tikhonova IG, Sum CS, Neumann S, Thomas CJ, Raaka BM, Costanzi S, Gershengorn MC (2007). Bidirectional, iterative approach to the structural delineation of the functional “Chemoprint” in GPR40 for agonist recognition. J Med Chem.

[14] Sum CS, Tikhonova IG, Neumann S, Engel S, Raaka BM, Costanzi S, Gershengorn MC (2007). Identification of residues important for agonist recognition and activation in GPR40. J Biol Chem.

[15] Tikhonova IG, Sum CS, Neumann S, Engel S, Raaka BM, Costanzi S, Gershengorn MC (2008). Discovery of novel Agonists and antagonists of the free fatty acid receptor 1 (FFAR1) using virtual screening. J Med Chem.

[16] Srivastava A, Yano J, Hirozane Y, Kefala G, Gruswitz F, Snell G, Lane W, Ivetac A, Aertgeerts K, Nguyen J, Jennings A, Okada K (2014). High-resolution structure of the human GPR40 receptor bound to allosteric agonist TAK-875. Nature.

[17] Sum CS, Tikhonova IG, Costanzi S, Gershengorn MC (2009). Two Arginine-Glutamate Ionic Locks Near the Extracellular Surface of FFAR1 Gate Receptor Activation. J Biol Chem.

[18] Zhang C, Srinivasan Y, Arlow DH, Fung JJ, Palmer D, Zheng Y, Green HF, Pandey A, Dror RO, Shaw DE, Weis WI, Coughlin SR, Kobilka BK (2012). High-resolution crystal structure of human protease-activated receptor 1. Nature.

[19] Yabuki C, Komatsu H, Tsujihata Y, Maeda R, Ito R, Matsuda-Nagasumi K, Sakuma K, Miyawaki K, Kikuchi N, Takeuchi K, Habata Y, Mori M (2013). A Novel Antidiabetic Drug, Fasiglifam/TAK-875, Acts as an Ago-Allosteric Modulator of FFAR1. PLoS One.

[20] Schrödinger, LLC, New York, NY, USA: Glide 6.5. 2014.

[21] Schrodinger, LLC, New York, NY, USA: Induced Fit Docking protocol 2014–4, Glide version 6.4, Prime version 3.7. 2014.

[22] Christiansen E, Hansen SV, Urban C, Hudson BD, Wargent ET, Grundmann M, Jenkins L, Zaibi M, Stocker CJ, Ullrich S, Kostenis E, Kassack MU, Milligan G, Cawthorne MA, Ulven T (2013). Discovery of TUG-770: A Highly Potent Free Fatty Acid Receptor 1 (FFA1/GPR40) Agonist for Treatment of Type 2 Diabetes. ACS Med Chem Lett.

[23] Lin DC, Guo Q, Luo J, Zhang J, Nguyen K, Chen M, Tran T, Dransfield PJ, Brown SP, Houze J, Vimolratana M, Jiao XY, Wang Y, Birdsall NJ, Swaminath G (2012). Identification and pharmacological characterization of multiple allosteric binding sites on the free fatty acid 1 receptor. Mol Pharmacol.

[24] Luo J, Swaminath G, Brown SP, Zhang J, Guo Q, Chen M, Nguyen K, Tran T, Miao L, Dransfield PJ, Vimolratana M, Houze JB, Wong S, Toteva M, Shan B, Li F, Zhuang R, Lin DC (2012). A potent class of GPR40 full agonists engages the enteroinsular axis to promote glucose control in rodents. PLoS One.

[25] Hudson BD, Due-Hansen ME, Christiansen E, Hansen AM, Mackenzie AE, Murdoch H, Pandey SK, Ward RJ, Marquez R, Tikhonova IG, Ulven T, Milligan G (2013). Defining the molecular basis for the first potent and selective orthosteric agonists of the FFA2 free fatty acid receptor. J Biol Chem.

[26] Stoddart LA, Smith NJ, Jenkins L, Brown AJ, Milligan G (2008). Conserved polar residues in transmembrane domains V, VI, and VII of free fatty acid receptor 2 and free fatty acid receptor 3 are required for the binding and function of short chain fatty acids. J Biol Chem.

[27] Hudson BD, Tikhonova IG, Pandey SK, Ulven T, Milligan G (2012). Extracellular ionic locks determine variation in constitutive activity and ligand potency between species orthologs of the free fatty acid receptors FFA2 and FFA3. J Biol Chem.

[28] Swaminath G, Jaeckel P, Guo Q, Cardozo M, Weiszmann J, Lindberg R, Wang Y, Schwandner R, Li Y (2011). Mutational analysis of G-protein coupled receptor--FFA2. Biochem Biophys Res Commun.

[29] Lee T, Schwandner R, Swaminath G, Weiszmann J, Cardozo M, Greenberg J, Jaeckel P, Ge H, Wang Y, Jiao X, Liu J, Kayser F, Tian H, Li Y (2008). Identification and functional characterization of allosteric agonists for the G protein-coupled receptor FFA2. Mol Pharmacol.

[30] Hudson BD, Christiansen E, Murdoch H, Jenkins L, Hojgaard Hansen A, Madsen OB, Ulven T, Milligan G (2014). Complex Pharmacology of Novel Allosteric Free Fatty Acid 3 Receptor Ligands. Mol Pharmacol.

[31] Smith NJ, Ward RJ, Stoddart LA, Hudson BD, Kostenis E, Ulven T, Morris JC, Trankle C, Tikhonova IG, Adams DR, Milligan G (2011). Extracellular loop 2 of the free fatty acid receptor 2 mediates allosterism of a phenylacetamide ago-allosteric modulator. Mol Pharmacol.

[32] Michino M, Abola E, Brooks CL, Dixon JS, Moult J, Stevens RC, GPCR Dock 2008 participants (2009). Community-wide assessment of GPCR structure modelling and ligand docking: GPCR Dock 2008. Nat Rev Drug Discov.

[33] Kufareva I, Rueda M, Katritch V, Stevens RC, Abagyan R, GPCR Dock 2010 participants (2011). Status of GPCR modeling and docking as reflected by community-wide GPCR Dock 2010 assessment. Structure.

[34] Kufareva I, Katritch V, Stevens RC, Abagyan R, Participants of GPCR Dock 2013 (2014). Advances in GPCR modeling evaluated by the GPCR Dock 2013 assessment: meeting new challenges. Structure.

[35] Worth CL, Kleinau G, Krause G (2009). Comparative sequence and structural analyses of G-protein-coupled receptor crystal structures and implications for molecular models. PLoS One.

[36] Bera I, Laskar A, Ghoshal N (2011). Exploring the structure of opioid receptors with homology modeling based on single and multiple templates and subsequent docking: a comparative study. J Mol Model.

[37] Watson SJ, Brown AJ, Holliday ND (2012). Differential signaling by splice variants of the human free fatty acid receptor GPR120. Mol Pharmacol.

[38] Hudson BD, Shimpukade B, Milligan G, Ulven T (2014). The molecular basis of ligand interaction at free fatty acid receptor 4 (FFA4/GPR120). J Biol Chem.

[39] Costanzi S (2012). Homology modeling of class a G protein-coupled receptors. Methods Mol Biol.

[40] Thompson JD, Higgins DG, Gibson TJ (1994). Clustal-W - Improving the Sensitivity of Progressive Multiple Sequence Alignment through Sequence Weighting, Position-Specific Gap Penalties and Weight Matrix Choice. Nucleic Acids Res.

[41] Henikoff S, Henikoff JG (1992). Amino acid substitution matrices from protein blocks. Proc Natl Acad Sci U S A.

[42] Soding J (2005). Protein homology detection by HMM-HMM comparison. Bioinformatics.

[43] Schrödinger, LLC, New York, NY, USA: Prime 3.0. 2011.

[44] Schrodinger, LLC, New York, NY, USA: Maestro 9.9. 2014.

[45] Sherman W, Day T, Jacobson MP, Friesner RA, Farid R (2006). Novel procedure for modeling ligand/receptor induced fit effects. J Med Chem.

[46] Sherman W, Beard HS, Farid R (2006). Use of an induced fit receptor structure in virtual screening. Chem Biol Drug Des.

[47] Humphrey W, Dalke A, Schulten K (1996). VMD: Visual molecular dynamics. J Mol Graph.

[48] Pettersen EF, Goddard TD, Huang CC, Couch GS, Greenblatt DM, Meng EC, Ferrin TE (2004). UCSF Chimera--a visualization system for exploratory research and analysis. J Comput Chem.

[49] Schrodinger, LLC, New York, NY, USA: SiteMap 3.3, 2014.

[50] Schrodinger, LLC, New York, NY, USA: MacroModel 10.6. 2014.

[51] Ballesteros JA, Weinstein H (1995). Modeling transmembrane helix contacts in GPCR. Biophys J.

