# Supplementary material for: Free fatty acid receptors: structural models and elucidation of ligand binding interactions
Source: BMC Struct Biol. 2015 Sep 7;15:16. doi: 10.1186/s12900-015-0044-2 (PMC4561419; doi:10.1186/s12900-015-0044-2)
Supplement: Additional file 1: — The binding cavities in the FFA1 crystal structure (A), rhodopsin (B) and β2 adrenergic receptor (C) based FFA1 homology models. (PDF 416 kb) [file 12900_2015_44_MOESM1_ESM.pdf]

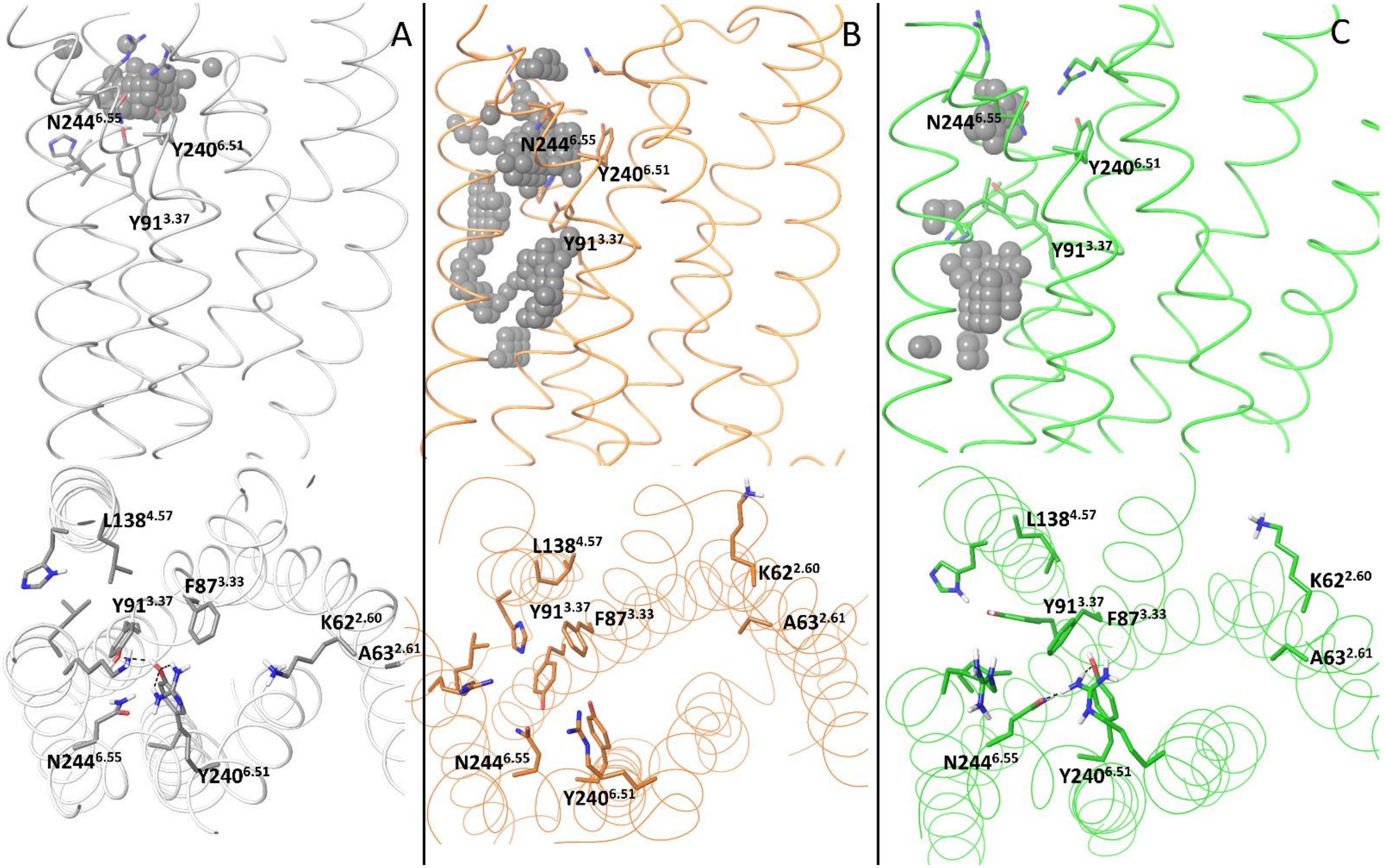

Additional Figure 1S. The binding cavities in the FFA1 crystal structure (A), rhodopsin (B) and β2 adrenergic receptor (C) based FFA1 homology models. The upper panel shows SiteMap (SiteMap, version 3.3, Schrodinger, LLC, New York, NY, 2014) results for the region involving helices 3-7. The lower panel shows the binding cavity from the top view. The key residues for agonist binding from mutagenesis and residues at positions 2.60 and 2.61 are visualized. The top view highlights the distant position of tyrosines at positions 3.37 and 6.51 in the homology models, which opens up the deeper cavity inside the helical bundle.
