# Supplementary material for: Free fatty acid receptors: structural models and elucidation of ligand binding interactions
Source: BMC Struct Biol. 2015 Sep 7;15:16. doi: 10.1186/s12900-015-0044-2 (PMC4561419; doi:10.1186/s12900-015-0044-2)
Supplement: Additional file 2: — The hydrogen bonding network involving water molecules in FFA1. (PDF 269 kb) [file 12900_2015_44_MOESM2_ESM.pdf]

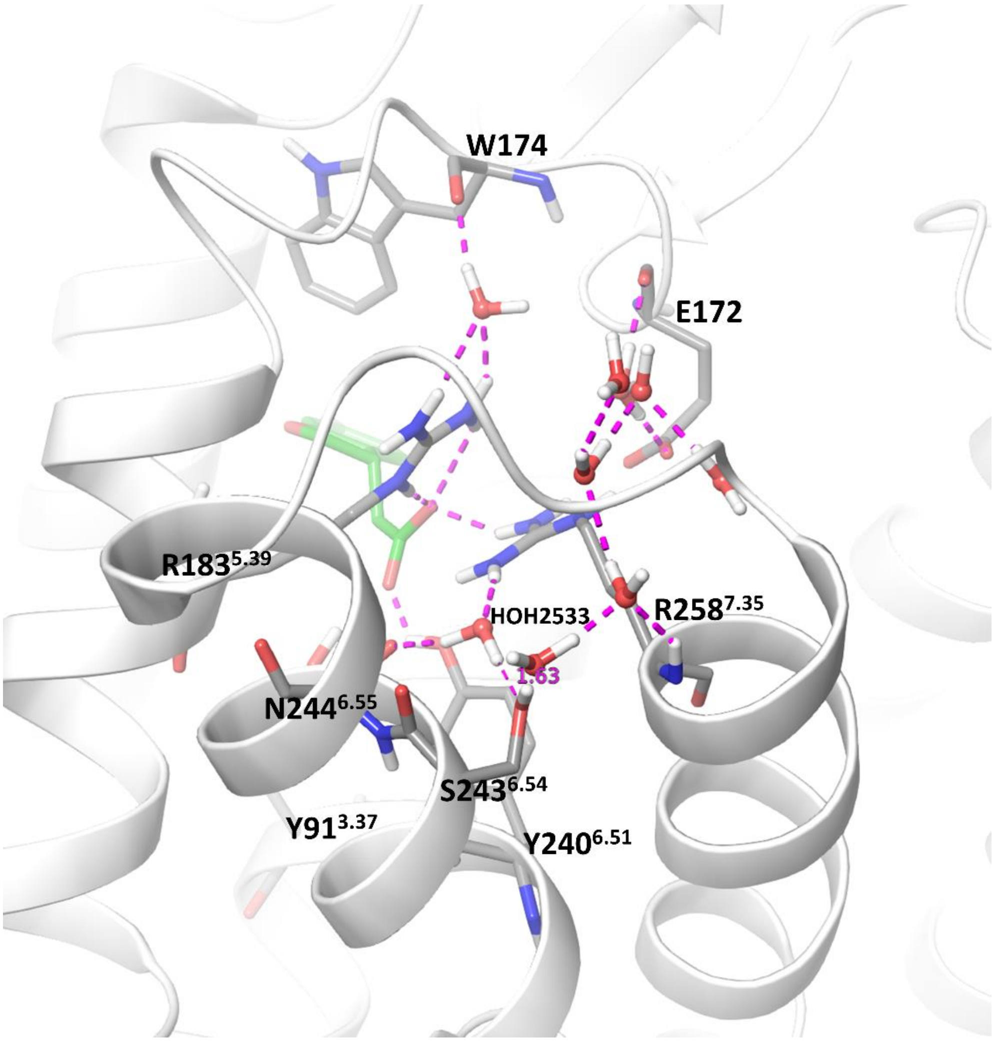

Additional Figure 2S. The hydrogen bonding network involving water molecules in FFA1 (PDB code: 4PHU). Hydrogen atoms are added to the crystal structure and minimized using a standard protocol of the protein preparation utility of Maestro 9.9.
