# Supplementary figures and images for: Free fatty acid receptors: structural models and elucidation of ligand binding interactions
Source: BMC Struct Biol. 2015 Sep 7;15:16. doi: 10.1186/s12900-015-0044-2 (PMC4561419; doi:10.1186/s12900-015-0044-2)

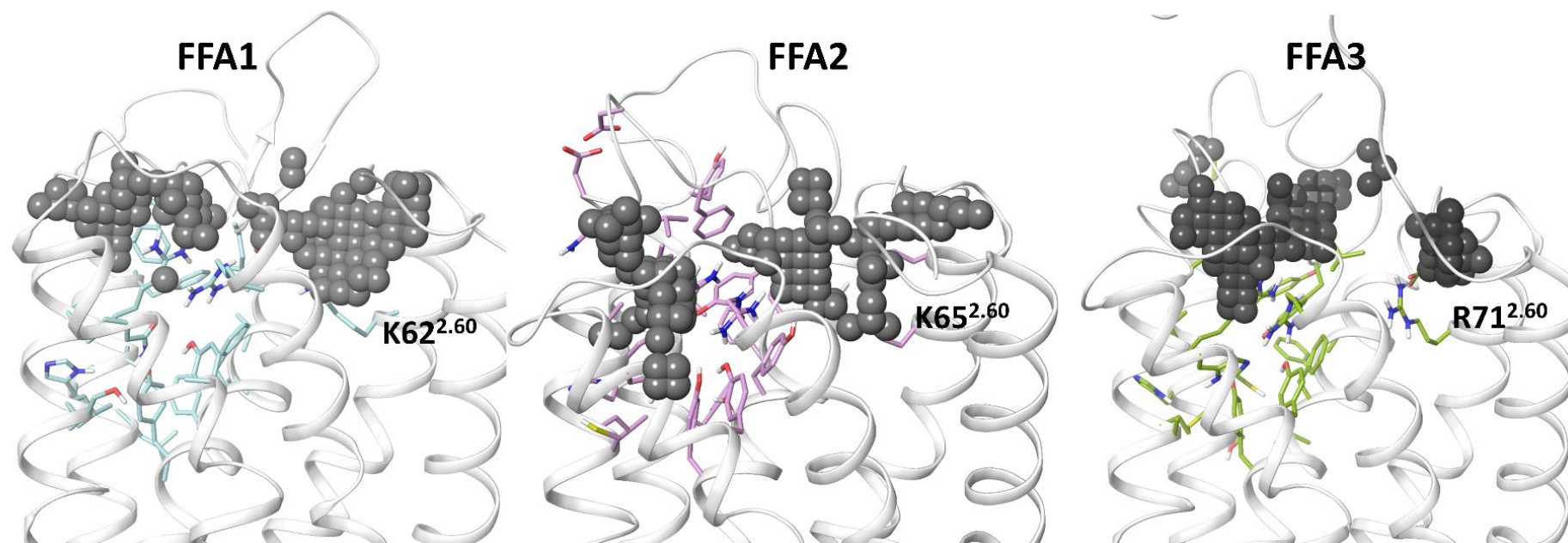

**Additional Figure 3S.** The location of putative allosteric sites at FFA1-3.

Supplement: Additional file 3: — The location of putative allosteric sites at FFA1-3. (PDF 310 kb) [file 12900_2015_44_MOESM3_ESM.pdf]
