# Supplementary material for: Free fatty acid receptors: structural models and elucidation of ligand binding interactions
Source: BMC Struct Biol. 2015 Sep 7;15:16. doi: 10.1186/s12900-015-0044-2 (PMC4561419; doi:10.1186/s12900-015-0044-2)
Supplement: Additional file 4: — The superimposition of the FFA1 crystal structure and a characteristic conformation of FFA1 from conformational search of the FFA1 crystal structure without the ligand. (PDF 309 kb) [file 12900_2015_44_MOESM4_ESM.pdf]

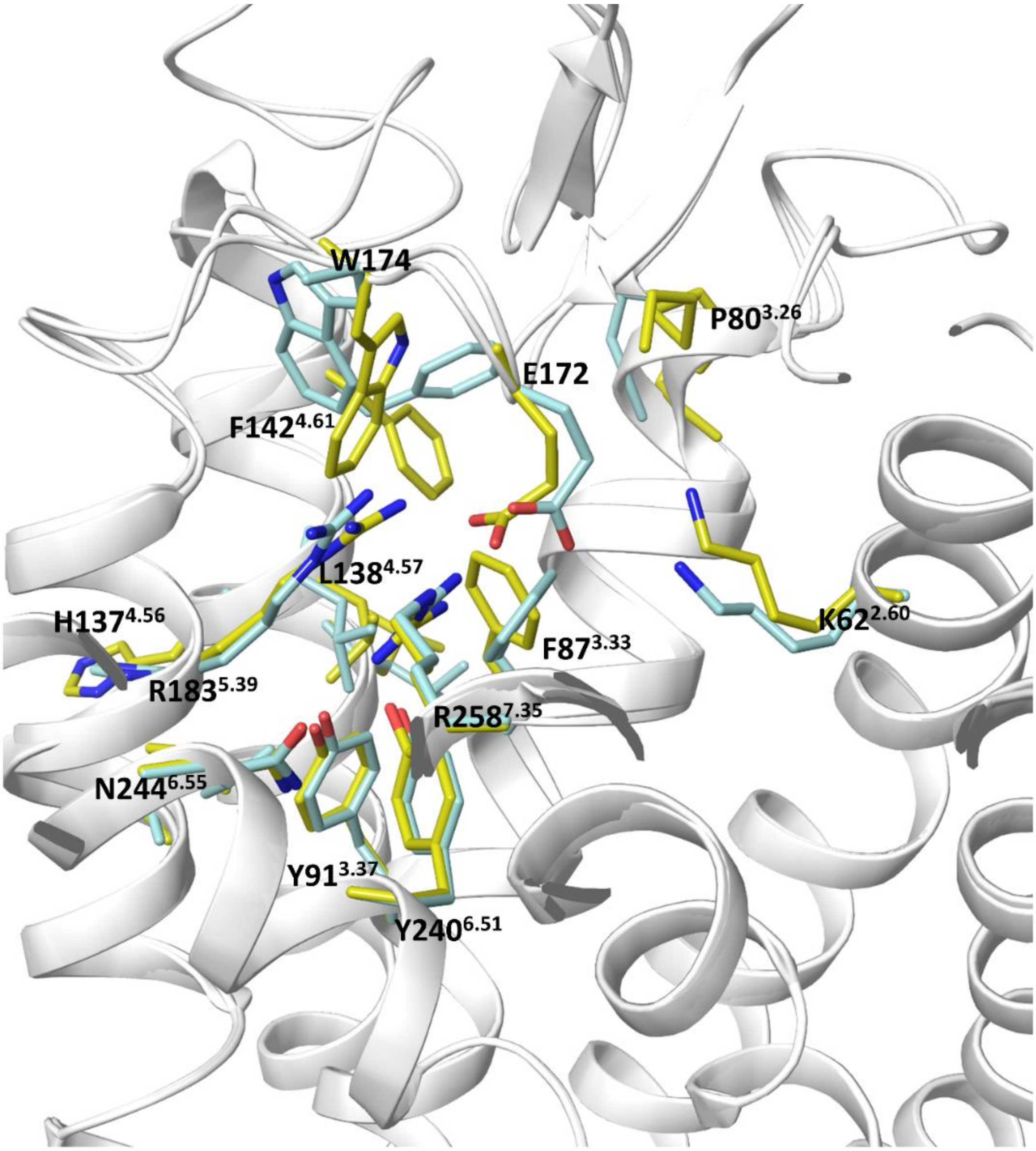

Additional Figure 4S. The superimposition of the FFA1 crystal structure and a characteristic conformation of FFA1 from conformational search of the FFA1 crystal structure without the ligand. The figure shows changes in the conformation of F87$^{3.33}$, F142$^{4.61}$ and W174^{EL2}, which close the interhelical space between helices 3 and 4.
