# Supplementary material for: Free fatty acid receptors: structural models and elucidation of ligand binding interactions
Source: BMC Struct Biol. 2015 Sep 7;15:16. doi: 10.1186/s12900-015-0044-2 (PMC4561419; doi:10.1186/s12900-015-0044-2)
Supplement: Additional file 5: — Agonist binding at the FFA1 crystal structure. (PDF 415 kb) [file 12900_2015_44_MOESM5_ESM.pdf]

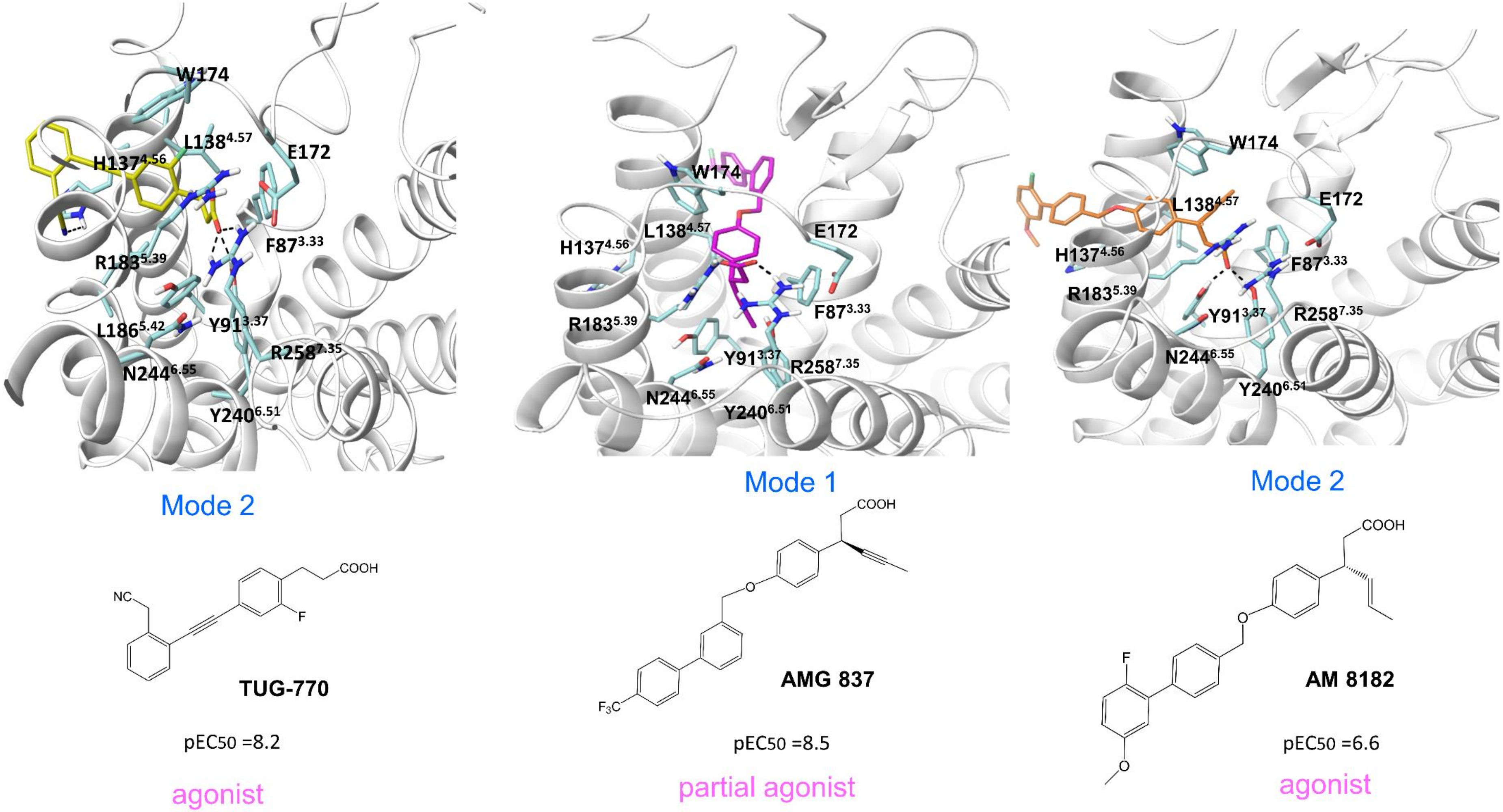

Additional Figure 5S. Agonist binding at the FFA1 crystal structure.
