# Supplementary material for: Free fatty acid receptors: structural models and elucidation of ligand binding interactions
Source: BMC Struct Biol. 2015 Sep 7;15:16. doi: 10.1186/s12900-015-0044-2 (PMC4561419; doi:10.1186/s12900-015-0044-2)
Supplement: Additional file 7: — Sequence alignment of the free fatty acid receptors and 25 GPCRs with available crystal structures and HHpred server predictions of a homologous relationship. (PDF 329 kb) [file 12900_2015_44_MOESM7_ESM.pdf]

```
                         *       20         *       40         *       60         *       80         *      100         *      120
FFAR1_HUMA : ---------MDLPPQLSFGLYVAAFALGFPLNVLAIRGATAHARLR-LTPSLVYALNLGCSDLLLTVSLPLKAVEALASGAWPLPASLCPVFAVAHFFPLYAGGGFLAALSAGRYLGAAFPIGYQAFRR : 119
FFAR2_HUMA : -------MLPDWKSSLILMAYIIIFLTGLPANLLALRAFVGRIRQPQPAPVHILLLSLTLADLLLLLLLPFKIIEAASNFRWYLPKVVCALTSFGFYSSIYCSTWLLAGISIERYLGVAFPVQYKLSRR : 122
FFAR3_HUMA : MDTGPDQSYFSGNHWFVFSVYLLTFLVGLPLNLLALVVFVGKLQR-RPVAVDVLLLNLTASDLLLLLFLPFRMVEAANGMHWPLPFILCPLSGFIFFTTIYLTALFLAAVSIERFLSVAHPLWYKTRPR : 128
2rh1_ADRB2 : ---------DEVWVVGMGIVMSLIVLAIVFGNVLVITAIAKFERL--QTVTNYFITSLACADLVMGLAVPFGAAHILMK-MWTFGNFWCEFWTSIDVLCVTASIETLCVIAVDRYFAITSPFKYQSLLT : 117
2z73_rho   : ---------PDAVYYSLGIFIGICGIIGCGGNGIVIYLFTKTKSL--QTPANMFIINLAFSDFTFSLVFPLMTISCFLK-KWIFGFAACKVYGFIGGIFGFMSIMTMAMISIDRNVIGRPMAASKKMS : 117
3odu_CXCR4 : --------NANFNKIFLPTIYSIIFLTGIVGNGLVILVMGYQKKL--RSMTDKYRLHLSVADLLFVITLPFWAVDAVA--NWYFGNFLCKAVHVIYTVNLYSSVWILAFISIDRYLAIVHATNSQRPRK : 117
3pbl_D3    : --------SQARPHAYYALSYCALILAIVFGNGLVCMAVLKERAL--QTTTNYLVVSLAVADLLVATLMPWVVYLEVTGGVWNFSRICCDVFVTLDVMMCTASIWNLCAISIDRYTAVVMPVHYTGQSS : 119
3rze_H1     : --------MASPQLMPLVVVLSTICLVTVGLNLLVLYAVRSERKL--HTVGNLYIVSLSVADLIVGAVMPMNILYLLMS-KWSLGRPLCLFWLSMDYVASTASIFSVFILCIDRYRSVQQPLRYLKYRT : 118
3uon_ACM2   : ---------KTFEVVFIVLVAGSLSLVTIIGNILVMVSIKVNRHL--QTVNNYFLFSLACADLIIGVFMNLYTLYTVIG-YWPLGPVVCDLWLALDYVVSNASVMNLLIISFDRYFCVTKPLTYPVKRT : 117
3v2y_S1P    : --------DKENSIKLTSVVFILICCFIILENIFVLLTIWKTKKF--HRPMYYFIGNLALSDLLAGVAYTANLLLSGAT-TYKLTPAQWFLREGSMFVALSASVFSLLAIAIERYITML-KMKLHNGSN : 117
3vw7_PAR1   : --------TSSWLTLFVPSVYTGVFVVSLPLNIMAIVVFILKMKV--KKPAVVYMLHLATADVLFVSVLPFKISYYFSGSDWQFGSELCRFVTAAFYCNMYASILLMTVISIDRFLAVVYPMQSLSWRT : 119
4bvn_ADRB1 : -------LLSQQWEAGMSLLMALVVLLIVAGNVLVIAAIGSTQRL--QTLTNLFITSLACADLVVGLLVVPFGATLVVRG-TWLWGSFLCELWTSLDVLCVTASVETLCVIAIDRYLAITSPFRYQSLMT : 119
4dkl_OPRM  : --------PSMVTAITIMALYSIVCVVGLFGNFLVMYVIVRYTKM--KTATNIYIFNLALADALATSTLPFQSVNYLMG-TWPFGNILCKIVISIDYYNMFTSIFTLCTMSVDRYIAVCHPVKALDFRT : 118
4ea3_OPRX  : --------LPLGLKVTIVGLYLAVCVGGLLGNCLVMYVILRHTKM--KTATNIYIFNLALADTLVLLTLPFQGTDILLG-FWPFGNALCKTVIAIDYYNMFTSTFTLTAMSVDRYVAICHPIRALDVRT : 118
4eiy_A2A   : --------PPIMGSSVYITVELAIAVLAILGNVLVCWAVWLNSNL--QNVTNYFVVSLAAADIAVGVLAIPFAITIST---GFCAACHGCLFIACFVLVLTQSSIFSLLAIAIDRYIAIRIPLRYNGLVT : 116
4grv_NTSR1 : --------TDIYSKVLVTAIYLALFVVGTVGNSVTLFTLARKKSL--QSTVHYHLGSLALSDLLILLLMPVELYNFIVHHPWAFGDAGCRGYYFLRDACTYATALNVASLSVARYLAICHPFKAKTLMS : 119
4iar_5HT1B : ---------SLPWKVLLVMLLALITLATTLSNAFVIATVYRTRKL--HTPANYLIASLAVTDLLVSILMPISTMYTVTG-RWTLGQVVCDFWLSSDITCCTASIWHLCVIALDRYWAITDAVEYSAKRT : 117
4ib4_5HT2B : --------EQGNKLHWAALLILMVIIPTIGGNTLVILAVSLEKKL--QYATNYFLMSLAVADLLVGLFVMPIALLTIMFEAMWPLPLVLCPAWLFLDVLFSTASIWHLCAISVDRYIAIKKPIQANQYNS : 119
4mbs_CCR5  : --------VKQIAARLLPPLYSLVFIFGFVGNMLVILILINYKRL--KSMTDIYLLNLAISDLFFLLTVPFWAHYAA--AQWDFGNTMCQLLTGLYFIGFFSGIFFIILLTIDRYLAVVHAVFALKART : 117
4n6h_OPRD  : --------SSLALAIAITALYSAVCAVGLLGNVLVMFGIVRYTKM--KTATNIYIFNLALADALATSTLPFQSAKYLME-TWPFGELLCKAVLSIDYYNMFTSIFTLTMMSVDRYIAVCHPVKALDFRT : 118
4phu_FFA1  : --------TMDLPPQLSFGLYVAAFALGFPLNVLAIRGATAHARL-RLTPSAVYALNLGCSDLLLTVSLPLKAVEALASGAWPLPASLCPVFAVAHFAPLYAGGGFLAALSAARYLGAAFPIGYQAFRR : 120
4pxz_P2Y12 : --------DYKITQVLFPLLYTVLFFVGLITNGLAMRIFFQIRSK--SNFI-IFLKNTVISDLLMILTFPFKILSDAKLGTGPLRTFVCQVTSVIFYFTMYISISFLGLITIDRYQKTTRPFKTSNPKN : 118
4s0v_OX2   : --------HPKEYEWVLIAGYIIVFVVALIGNVLVCVAVWKNHHM--RTVTNYFIVNLSLADVLVTITCLPATLVVDITE-TWFFGQSLCKVIPYLQTVSVSVSVLTLSCIALDRWYAICHPIMFKS--T : 116
4u15_ACM3  : --------HTIWQVVFIAFLTGFLALVTIIGNILVIVAFKVNKQL--KTVNNYFLLSLACADLIIGVIMNLFTTYIIMN-RWALGNLACDLWLSIDYVASNASVMNLLVISFDRYFSITRPLTYRAKRT : 118
4xnv_P2Y1  : --------KTGFQFYYLPAVYILVFIIGFLGNSVAIWMFVFHMKP--WSGISVYMFNLALADFLYVLTLPALIFYYFNKTDWIFGDAMCKLQRFIFHVNLYGSILFLTCISAHRYSGVVYPLKSLGRLK : 119
4xt1_CX3CL : ---------LNQSKPVTLFLYGVVFLFGSIGNFLVIFTITWRRRI--QCSGDVYFINLAAADLLFVCTLPLWMQYL--LDHNSLASVPCTLLTACFYVAMFASLCFITEIALDRYYAIVY----MRYRP : 112
4yay_AT1R  : --------RHNYIFVMIPTLYSIIFVVGIFGNSLVVIVIYFMKL--KTVASVFLLNLALADLCFLLTLPLWAVYTAMEYRWPFGNYLCKIASASVSFNLYASVFLLTCLSIDRYLAIVHPMKSRLRRT : 119
4z35_LPA1  : --------EWNTVSKLVMGLGITVCIFIMLANLLVMVAIYVNRRF--HFPIYYLMANLAAADFFAGLAYFYLMFNTGPN-TRRLTVSTWLLRQGLIDTSLTASVANLLAIAIERHITVFR-MQLHTRMS : 117

                                1.50                                 2.50                                                     3.50

                 *       140        *       160        *       180        *       200        *       220        *       240        *    2
FFAR1_HUMA : PCYSWGVCAAIWALVLCHLGLVFGLEAPGGWLDHSNTSLGINTPVNGSPVCLEAWDPASAG---PARFSLSLLLFFLPLAITAFCYVGCLRALARSG-LTHRRKLRAAWVAGGALLTLLLCVGPYNASN : 244
FFAR2_HUMA : PLYGVIAALVAWVMSFGHCTIVIIVQYLNTTEQVR---------SGNEITCYENFTDNQLDVVLPVRLELCLVLFFIPMAVTIFCYWRFVWIMLSQPLVGAQRRRRAVGLAVVTLLNFLVCFGPYNVSH : 242
FFAR3_HUMA : LGQAGLVSVACWLLASAHCSVVYVIEFSGDISHS----------QGTNGTCYLEFRKDQLAILLPVRLEMAVVLFVVPLIITSYCYSRLVWILGRGG--SHRRQRRVAGLLAATLLNFLVCFGPYNVSH : 245
2rh1_ADRB2 : KNKARVIILMVWIVSGLTSFLPIQMHWYRATH-------------QEAINCYAEETCCDFFTNQAYAIASSIVSFYVPLVIMVFVYSRVFQEAKRQLNKFCLKEHKALKTLGIIMGTFTLCWLPFFIVN : 233
2z73_rho    : HRRAFIMIIFVWLWSVLWAIGPIFGWGAYTLE-------------GVLCNCSFDYISRDST-TRSNILCMFILGFFGPILIIFFCYFNIVMSVSRKAQAGANAEMRLAKISIVIVSQFLLSWSPYAVVA : 232
3odu_CXCR4 : LLAEKVVYVGVWIPALLLTIPDFIFANVSEA--------------DDRYICDRFYPNDLWVV--VFQFQHIMVGLILPGIVILSCYCIIISKLSHSYGSKGHQKRKALKTTVILILAFFACWLPYYIGI : 230
3pbl_D3     : CRRVALMITAVWVLAFAVSCPLLFGFNTT----------------GDPTVCSISNP--------DFVIYSSVVSFYLPFGVTVLVYARIYVVLKQRRRKNIFEREKKATQMVAIVLGAFIVCWLPFFLTH : 224
3rze_H1     : KTRASATILGAWFLSFLWVIPILGWNHFMQQTSV-----------RREDKCETDFYDVTW-----FKVMTAIINFYLPTLLMLWFYAKIYKAVRQHAYLHMNRERKAAKQLGFIMAAFILCWIPYFIFF : 231
3uon_ACM2   : TKMAGMMIAAAWVLSFILWAPAILFWQFIVGVRT-----------VEDGECYIQFFSNAA-----VTFGTAIAAFYLPVIIMTVLYWHISRASKSRINIFESREKKVTRTILAILLAFIITWAPYNVMV : 230
3v2y_S1P    : NFRLFLLISACWVISLILGGLPIMGWNCI----------------SALSSCSTVLPLYHKH-----YILFCTTVFTLLLLSIVILYCRIYSLVRTRNISRSSENVALLKTVIIVLSVFIACWAPLFILL : 225
3vw7_PAR1   : LGRASFTCLAIWALAIAGVVPLLLKEQTIQVPG------------LGITTCHDVLSETLEGYYAYYFSAFSAVFFFVPLIISTVCYVSIIRCLSSSANANRSKKSRALFLSAAVFCIFIICFGPTNVLL : 236
4bvn_ADRB1 : RARAKVIICTVWAISALVSFLPIMMHWWRDED-------------PQALKCYQDPGCCDFVTNRAYAIASSIISFYIPLLIMIFVALRVYREAKEQIRKIDMREHKALKTLGIIMGVFTLCWLPFFLVN : 235
4dkl_OPRM  : PRNAKIVNVCNWILSSAIGLPVMFMATTKYR--------------QGSIDCTLTFSHPTWYWENLLKICVFIFAFIMPVLIITVCYGLMILRLKSVRNEKDRNLRRITRMVLVVVAVFIVCWTPIHIYV : 233
4ea3_OPRX  : SSKAQAVNVAIWALASVVGVPVAIMGSAQVE--------------DEEIECLVEIPTPQDYWGPVFAICIFLFSFIVPVLVISVCYSLMIRRLRGVSREKDRNLRRITRLVLVVVAVFVGCWTPVQVFV : 233
4eiy_A2A   : GTRAKGIIAICWVLSFAIGLTPMLGWNNCGQPKEG----------KNHSQGCGEGQVAEDVVPMNYMVYFNFFACVLVPLLLMLGVYLRIFLAARRQLADTLQKEVHAAKSLAIIVGLFALCWLPLHIIN : 236
4grv_NTSR1 : RSRTKKFISAIWLASALLAIPMLFTMGLQNRSH------------PGGLVCTPIVDTATVK---VVIQVNTFMSFLFPMLVISILNTVIANKLTVMVNIFVQALRHGVLVARAVVIAFVVCWLPYHVRR : 233
4iar_5HT1B : PKRAAVMIALVWVFSISISLPPFF--WRQAKAE------------EEVSECVVNTDHI------LYTVYSTVGAFYFPTLLLIALYGRIYVEARSRIADLEARERKATKTLGIILGAFIVCWLPFFIIS : 226
4ib4_5HT2B : RATAFIKITVVWLISIGIAIPVPIKGIETDVDN------------PNNITCVLTK-----ERFGDFMLFGSLAAFFTPLAIMIVTYFLTIHALQKKAADLESNEQRASKVLGIVFFLFLLMWCPFFITN : 231
4mbs_CCR5  : VTFGVVTSVITWVVAVFASLPNIIFTRSQKE--------------GLHYTCSSHFPYSQYQWKNFQTLKIVILGLVLPLLVMVICYSGILKTLLRMKKEEEKKRHRDVRLIFTIMIVYFLFWAPYNIVL : 232
4n6h_OPRD  : PAKAKLINICIWVLASGVGVPIMVMAVTRPR--------------DGAVVCMLQFPSPSWYWDTVTKICVFLFAFVVPILIITVCYGLMLLRLRSVSKEKDRSLRRITRMVLVVVGAFVVCWAPIHIFV : 233
4phu_FFA1  : PCYSWGVCAAIWALVLCHLGLVFGLEWLDHSNTPV----------NGSPVCLEAWDPASAG---PARFSLSLLLFFLPLAITAFCFVGCLRALARGSNIFHRRKLRAAWVAGGALLTLLLCVGPYNASN : 236
4pxz_P2Y12 : LLGAKILSVVIWAFMFLLSLPNMILTNRQPRD-------------KNVKKCSFLKSEFGLV-WHEIVNYICQVIFWINFLIVIVCYTLITKELYRSYVRGKVPRKKVNVKVFIIIAVFFICFVPFHFAR : 233
```

```
4s0v_OX2    : AKRARNSIVIIWIVSCIIMIPQAIVMECSTVNKT-----------TLFTVCDERWGGEIYPK--MYHICFFLVTYMAPLCLMVLAYLQIFRKLWCRQGIDIRARRKTARMLMVVLLVFAICYLPISILN : 232
4u15_ACM3   : TKRAGVMIGLAWVISFVLWAPAILFWQYFVGKRT-----------VPPGECFIQFLSEP-----TITFGTAIAAFYMPVTIMTILYWRIYKETEKMNDAYLIKEKKAAQTLSAILLAFIITWTPYNIMV : 231
4xnv_P2Y1   : KKNAICISVLVWLIVVVAISPILFYSGTGVRK-------------NKTITCYDTTSDEYLRSYFIYSMCTTVAMFCVPLVLILGCYGLIVRALIYKMKKEEPLRRKSIYLVIIVLTVFAVSYIPFHVMK : 235
4xt1_CX3CL  : VKQACLFSIFWWIFAVIIAIPHFMVVT------------------KKDNQCMTDYDYLEVSYPIILNVELMLGAFVIPLSVISYCYYRISRIVAVS---QSRHKGRIVRVLIAVVLVFIIFWLPYHLTL : 220
4yay_AT1R   : MLVAKVTCIIIWLLAGLASLPAIIHRNVFFIEN------------TNITVCAFHYESQNSTLPIGLGLTKNILGFLFPFLIILTSYTLIWKALKKAYEINKPRNDDIFKIIMAIVLFFFFSWIPHQIFT : 236
4z35_LPA1   : NRRVVVVIVVIWTMAIVMGAIPSVGWNCI----------------CDIENCSNMAPLYSDS-----YLVFWAIFNLVTFVVMVVLYAHIFGYVADLYLRNRDTMMSLLKTVVIVLGAFIICWTPGLVLL : 225
                         4.50                                                    5.50                                     6.60

               60         *        280         *        300         *        320         *        340         *        360
FFAR1_HUMA  : VASFLYPNLGGSWRKLGLITGAWSVVLNPLVTGYLGRGPGLKTVCAARTQGGKSQK---------------------------------------------- : 300
FFAR2_HUMA  : LVGYHQRKSP-WWRSIAVVFSSLNASLDPLLFYFSSSVVRRAFGRGLQVLRNQGSSLLGRRGKDTAEGTNEDRGVGQGEGMPSSDFTTE------------- : 330
FFAR3_HUMA  : VVGYICGESP-AWRIYVTLLSTLNSCVDPFVYYFSSSGFQADFHELLRRLCGLWGQWQQESSMELKEQKGGEEQRADRPAERKTSEHSQGCGTGGQVACAES : 346
2rh1_ADRB2  : IVHVIQDNRK-EVYILLNWIGYVNSGFNPLIYC-RSPDFRIAFQELLCLRRSSLKAYGNGYSSNGNTGEQSG------------------------------ : 303
2z73_rho    : LLAQFGPLTP-YAAQLPVMFAKASAIHNPMIYSVSHPKFREAISQTFPWVCCQFDDKETEDDKDAETEIPAGESSDAAPSADAAQMKEM------------- : 320
3odu_CXCR4  : SIDSFENTVH-KWISITEALAFFHCCLNPILYAFLGAKFKTSAQHALTSGRPL------------------------------------------------- : 282
3pbl_D3     : VLNTHCQTSP-ELYSATTWLGYVNSALNPVIYTTFNIEFRKAFLKILSCGRPLE------------------------------------------------ : 277
3rze_H1     : MVIAFCKNNE-HLHMFTIWLGYINSTLNPLIYPLCNENFKKTFKRILHIRSGEN------------------------------------------------ : 284
3uon_ACM2   : LINTFCAPPN-TVWTIGYWLCYINSTINPACYALCNATFKKTFKHLLMCHYKNIGA---------------------------------------------- : 285
3v2y_S1P    : LLDVGCKDIL-FRAEYFLVLAVLNSGTNPIIYTLTNKEMRRAFIRIMGRPLEVLFQGPHHHHHHHHHHDYKDDDDK-------------------------- : 300
3vw7_PAR1   : IAHYSFLSAY-FAYLLCVCVSSISCCIDPLIYYYASSECQRYVYSILCCKESSDPSCYHHHHHHHHHH---------------------------------- : 303
4bvn_ADRB1  : IVNVFNRDPK-WLFVAFNWLGYANSAMNPIILC-RSPDFRKAFKRLLAFPRKADRRLHHHHHH--------------------------------------- : 296
4dkl_OPRM   : IIKALITIPE-TTFQTVSWLGYTNSCLNPVLYAFLDENFKRCFREFCIPTSSTIEQP--------------------------------------------- : 289
4ea3_OPRX   : LAQGLGVQPS-SETAVCTALGYVNSCLNPILYAFLDENFKACFRKFCCASALGRPLEVLFQGPHHHHHHHHHH----------------------------- : 305
4eiy_A2A    : CFTFFCPDPL-WLMYLAIVLSHTNSVVNPFIYAYRIREFRQTFRKIIRSHVLRQQEPFKAHHHHHHHHHH-------------------------------- : 305
4grv_NTSR1  : LMFCYISDEQ-WFYMLTNALAYASSAINPILYNLVSANFRQVFLSTLACLCPGWRHRRKAHHHHHHHHHHGG------------------------------ : 304
4iar_5HT1B  : LVMPICKDHL-AIFDFFTWLGYLNSLINPIIYTMSNEDFKQAFHKLIRFKCTS------------------------------------------------- : 278
4ib4_5HT2B  : ITLVLCDSLQ-MLLEIFVWIGYVSSGVNPLVYTLFNKTFRDAFGRYITCNYRATKSVGRPLE---------------------------------------- : 292
4mbs_CCR5   : LLNTFSNRLD-QAMQVTETLGMTHCCINPIIYAFVGEEFRNYLLVFFQKHIAKRFCKCCSIFQQEAPERASSVYTRSTGEQEISVGLGR------------- : 320
4n6h_OPRD   : IVWTLVDIVV-AALHLCIALGYANSSLNPVLYAFLDENFKRCFRQLCRKPCG-------------------------------------------------- : 284
4phu_FFA1   : VASFLYPNLG-SWRKLGLITGAWSVVLNPLVTGYLGRGPGLKTVCAARTQGGKSQKAENLYFQGHHHHHHHH------------------------------ : 307
4pxz_P2Y12  : IPYTLSQTLF-YVKESTLWLTSLNACLNPFIYFFLCKSFRNSLISMLKCPNSATSLSQDNRKKEQDGGDPNEETPMGRP----------------------- : 311
4s0v_OX2    : VLKRVFGMFA-HWFTFSHWLVYANSAANPIIYNFLSGKFREEFKAAFSCCCLGVHHHHHHHHHH-------------------------------------- : 295
4u15_ACM3   : LVNTFCDSPK-TYWNLGYWLCYINSTVNPVCYALCNKTFRTTFKTLLLCQCDKRKRRKHHHHHHHHH----------------------------------- : 296
4xnv_P2Y1   : TMNLRADRVY-ATYQVTRGLASLNSCVNPILYFLAGDTFRRRLSRATRKASRRSEANLQSKSEDMTLNILPEFKQNGDTSL--------------------- : 315
4xt1_CX3CL  : FVDTLKRSLK-RALILTESLAFCHCCLNPLLYVFVGTKFRQELHCLLAEFRQRLFSRDVSWYHSMSFSRRSSPSRRETSSDTLSDEVCR------------- : 308
4yay_AT1R   : FLDVLIQLGD-TAMPITICIAYFNNCLNPLFYGFLGKKFKRYFLQLLKY----------------------------------------------------- : 284
4z35_LPA1   : LLDVCCPQVL-AYEKFFLLLAEFNSAMNPIIYSYRDKEMSATFRQILGRPLEVLFQGPHHHHHHHHHHDYKDDDDK-------------------------- : 300
                                      7.50
```

Additional Figure 7S. Sequence alignment of the free fatty acid receptors and 25 GPCRs with available crystal structures. The sequence alignment was built using the HHpred server (http://toolkit.tuebingen.mpg.de/hhpred). The sequence name starts with the pdb-code and ends with the receptor name.

N	Template	FFA1			FFA2			FFA3		
	PDB_name	probability	E-value	P-value	probability	E-value	P-value	probability	E-value	P-value
1	2rh1_ADRB2	100	4.90E-43	1.40E-47	100	5.00E-44	1.40E-48	100	7.10E-44	2.00E-48
2	2z73_RHODOPSIN	100	9.70E-41	2.70E-45	100	5.20E-42	1.50E-46	100	8.10E-43	2.30E-47
3	3odu_CXCR4	100	5.00E-43	1.40E-47	100	7.00E-43	2.00E-47	100	4.00E-43	1.10E-47
4	3pbl_D3	100	6.80E-42	1.90E-46	100	3.80E-42	1.10E-46	100	1.60E-42	4.60E-47
5	3rze_H1	100	9.70E-43	2.80E-47	100	1.60E-42	4.40E-47	100	2.10E-42	5.90E-47
6	3uon_ACM2	100	4.10E-43	1.10E-47	100	7.40E-43	2.10E-47	100	2.20E-43	6.20E-48
7	3v2y_S1P	100	4.50E-39	1.30E-43	100	1.80E-40	5.10E-45	100	2.90E-40	8.10E-45
8	3vw7_PAR1	100	2.20E-43	6.30E-48	100	3.00E-44	8.50E-49	100	1.60E-44	4.50E-49
9	4bvn_ADRB1	100	3.30E-44	9.40E-49	100	1.00E-44	2.90E-49	100	1.80E-44	5.10E-49
10	4dkl_OPRM	100	2.70E-43	7.70E-48	100	2.80E-43	8.00E-48	100	1.10E-43	3.20E-48
11	4ea3_OPRX	100	4.10E-40	1.20E-44	100	7.60E-41	2.10E-45	100	3.30E-41	9.40E-46
12	4eiy_A2A	100	9.20E-41	2.60E-45	100	6.60E-42	1.90E-46	100	1.30E-41	3.60E-46
13	4grv_NTSR1	100	2.70E-41	7.60E-46	100	4.20E-42	1.20E-46	100	1.10E-42	3.20E-47
14	4iar_5HT1B	100	2.80E-44	7.90E-49	100	4.40E-44	1.20E-48	100	2.20E-44	6.30E-49
15	4ib4_5HT2B	100	1.60E-41	4.60E-46	100	2.00E-41	5.60E-46	100	9.40E-42	2.70E-46
16	4mbs_CCR5	100	2.60E-42	7.50E-47	100	2.10E-43	6.10E-48	100	1.10E-43	3.10E-48
17	4n6h_OPRD	100	4.50E-40	1.30E-44	100	4.10E-40	1.20E-44	100	2.10E-40	6.10E-45
18	4phu_FFA1	100	5.10E-42	1.40E-46	100	7.90E-42	2.20E-46	100	3.30E-42	9.30E-47
19	4pxz_P2Y12	100	5.80E-44	1.60E-48	100	1.40E-44	3.90E-49	100	1.20E-45	3.30E-50
20	4s0v_OX2	100	1.20E-39	3.40E-44	100	2.80E-39	7.80E-44	100	1.40E-39	3.80E-44
21	4u15_ACM3	100	3.40E-42	9.70E-47	100	1.00E-42	2.90E-47	100	7.70E-43	2.20E-47
22	4xnv_P2Y1	100	1.40E-42	3.90E-47	100	3.60E-44	1.00E-48	100	3.00E-44	8.60E-49
23	4xt1_CX3CL	100	2.50E-39	7.10E-44	100	2.70E-41	7.50E-46	100	2.60E-42	7.30E-47
24	4yay_AT1R	100	6.20E-40	1.70E-44	100	1.30E-40	3.60E-45	100	5.00E-41	1.40E-45
25	4z35_LPA1	100	1.20E-38	3.30E-43	100	2.1E-40	5.9E-45	100	1.20E-40	3.40E-45

Additional Table 2S. HHpred server (http://toolkit.tuebingen.mpg.de/hhpred) predictions of a homologous relationship. Probability represents a homologous relationship; E-value is the average expected number of non-homologous proteins with a score higher than the one obtained for the database match. An E-value <<< 1 indicates statistical significance; and P-values is the area under the extreme value distribution. Receptor templates are shown with a pdb code and receptor name. The calculated values indicate a strong homologous relationship.
