# Supplementary material for: Free fatty acid receptors: structural models and elucidation of ligand binding interactions
Source: BMC Struct Biol. 2015 Sep 7;15:16. doi: 10.1186/s12900-015-0044-2 (PMC4561419; doi:10.1186/s12900-015-0044-2)
Supplement: Additional file 8: — Sequence identity (%) in the transmembrane helices of the FFA receptors and 25 GPCRs with available crystal structures. (PDF 343 kb) [file 12900_2015_44_MOESM8_ESM.pdf]

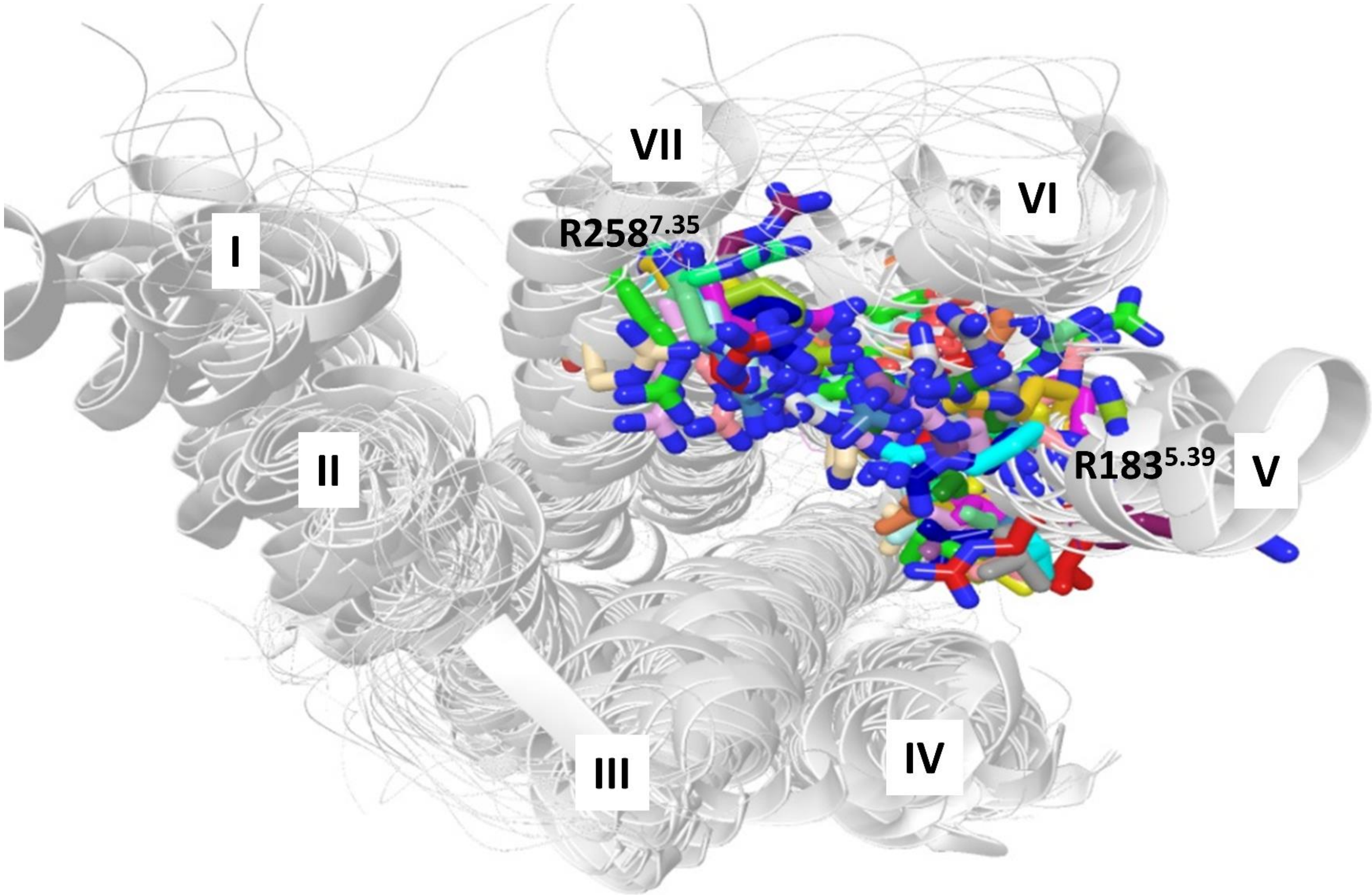

Additional Figure 8S. Superimposition of 25 FFA2 homology models built based on 25 GPCRs with an available crystal structure. Arginines at positions 5.39 and 7.35 are visualized.
