# Supplementary material for: Free fatty acid receptors: structural models and elucidation of ligand binding interactions
Source: BMC Struct Biol. 2015 Sep 7;15:16. doi: 10.1186/s12900-015-0044-2 (PMC4561419; doi:10.1186/s12900-015-0044-2)
Supplement: Additional file 9: — Superimposition of 25 FFA2 homology models built based on 25 GPCRs with an available crystal structure. (PDF 246 kb) [file 12900_2015_44_MOESM9_ESM.pdf]

Available receptor templates	FFA1 GPR40	FFA2 GPR43	FFA3 GPR41
**Amine Family**			
β adrenergic 1 ($β_1$)	18	18	15
β adrenergic 2 ($β_2$)	16	17	17
dopamine (D_3)	18	22	23
histamine1 (H_1)	16	19	18
muscarinic acetylcholine 2 (M_2)	16	22	21
muscarinic acetylcholine 3 (M_3)	15	21	21
serotonin ($5HT_{1B}$)	14	21	18
serotonin ($5HT_{2B}$)	22	22	19
**Peptide Family**			
angiotensin (AT_1)	**26**	22	25
chemokine ($CXCR_4$)	23	25	25
chemokine (CCR5)	24	23	27
chemokine (CX3CR1)	24	24	27
neurotensin (NTS_1)	16	21	22
nociceptin (NOP)	21	22	28
δ opioid (δ-OP)	21	22	24
κ opioid (κ-OP)	18	26	24
μ opioid (μ-OP)	19	22	25
orexin (OX_2)	19	20	23
protease-activated (PAR_1)	**26**	28	**33**
**Nucleotide Family**			
adenosine (A_{2A})	17	17	18
purine ($P2Y_{12}$)	20	**30**	26
purine ($P2Y_1$)	19	23	28
**Lysosphingolipid Family**			
lysophosphatidic acid (LPA_1)	13	19	19
sphingosine 1-phosphate ($S1P_1$)	16	17	17
**Free Fatty Acid Family**			
free fatty acid (FFA1)		**32**	**33**
**Rhodopsin** (rho, opsin)	16	22	22

Additional Table 1S. Sequence identity (%) in the transmembrane helices of the FFA receptors and 26 GPCRs with available crystal structures. High identity is highlighted in bold.
