# Supplementary material for: Free fatty acid receptors: structural models and elucidation of ligand binding interactions
Source: BMC Struct Biol. 2015 Sep 7;15:16. doi: 10.1186/s12900-015-0044-2 (PMC4561419; doi:10.1186/s12900-015-0044-2)
Supplement: Additional file 6: — Sequence alignment of the free fatty acid receptors. (PDF 235 kb) [file 12900_2015_44_MOESM6_ESM.pdf]

```
                    *        20         *        40         *        60         *
FFA1_h: ---------MDLPPQLSFGLYVAAFALGFPLNVLAIRGATAHARL-RLTPSLVYALNLGCSDLLLTVSLPLKAVEALA :  68
FFA2_h: -------MLPDWKSSLILMAYIIIFLTGLPANLLALRAFVGRIRQPQPAPVHILLLSLTLADLLLLLLLPFKIIEAAS :  71
FFA3_h: MDTGPDQSYFSGNHWFVFSVYLLTFLVGLPLNLLALVVFVGKLQR-RPVAVDVLLLNLTASDLLLLLFLPFRMVEAAN :  77
                               1.50                          2.50

              80         *       100         *       120         *       140         *
FFA1_h: SGAWPLPASLCPVFAVAHFFPLYAGGGFLAALSAGRYLGAAFPLGYQAFRRPCYSWGVCAAIWALVLCHLGLVFGLEA : 146
FFA2_h: NFRWYLPKVVCALTSFGFYSSIYCSTWLLAGISIERYLGVAFPVQYKLSRRPLYGVIAALVAWVMSFGHCTIVIIVQY : 149
FFA3_h: GMHWPLPFILCPLSGFIFFTTIYLTALFLAAVSIERFLSVAHPLWYKTRPRLGQAGLVSVACWLLASAHCSVVYVIEF : 155
                                            3.50                      4.50

               160         *       180         *       200         *       220         *
FFA1_h: PGGWLDHSNTSLGINTPVNGSPVCLEAWD---PASAGPARFSLSLLLFFLPLAITAFCYVGCLRALARSG-------L : 214
FFA2_h: LN---TTEQVRSGNE------ITCYENFTDNQLDVVLPVRLELCLVLFFIPMAVTIFCYWRFVWIMLSQPLVGAQRR- : 217
FFA3_h: SG---DISHS-QGTN------GTCYLEFRKDQLAILLPVRLEMAVVLFVVPLIITSYCYSRLVWILGRG--------G : 215
                                                           5.50

                240         *       260         *       280         *       300         *
FFA1_h: THRRKLRAAWVAGGALLTLLLCVGPYNASNVASFLYPNLGGSWRKLGLITGAWSVVLNPLVTGYLGRGPGLKTVCAAR : 292
FFA2_h: -----RRAVGLAVVTLLNFLVCFGPYNVSHLVGYHQRK-SPWWRSIAVVFSSLNASLDPLLFYFSSSVVRRAFGRGLQ : 289
FFA3_h: SHRRQRRVAGLLAATLLNFLVCFGPYNVSHVVGYICGE-SPAWRIYVTLLSTLNSCVDPFVYYFSSSGFQADFHELLR : 292
                              6.50                            7.50

                 320
FFA1_h: TQGGKSQK- : 300
FFA2_h: VLRNQGSSL : 298
FFA3_h: RLCGL---- : 297
```

Additional Figure 6S. Sequence alignment of the free fatty acid receptors.
